# Models of the human heart for biomedical research: Opportunities and challenges

**DOI:** 10.14814/phy2.70845

**Published:** 2026-04-09

**Authors:** Katrin Streckfuss‐Bömeke, Laura C. Zelarayán, Renate B. Schnabel, Nicolle Kränkel, Christoph Maack, Thomas Eschenhagen, Hannah E. Kappler, Ursula Klingmüller, Rafael Kramann, Axel Loewe, Hendrik Milting, Cristina E. Molina, Daniela Panáková, Bruno K. Podesser, Angelika Schnieke, Katrin Schröder, Thomas Seidel, Samuel Sossalla, Callum Zgierski‐Johnston, Wolfram‐Hubertus Zimmermann, Eva A. Rog‐Zielinska, Peter Kohl

**Affiliations:** ^1^ Institute of Pharmacology and Toxicology University of Würzburg Würzburg Germany; ^2^ Cardiology and Angiology, Medical Clinic I Justus‐Liebig‐University Giessen Germany; ^3^ Institute of Pharmacology and Toxicology University Medical Center Göttingen Germany; ^4^ German Centre for Cardiovascular Research (DZHK), Partner Site Lower Saxony Göttingen Germany; ^5^ Experimental Cardiology, Cardiology and Angiology, Medical Clinic I Justus‐Liebig‐University Giessen Germany; ^6^ Department of Cardiology University Heart & Vascular Center Hamburg, University Medical Center Hamburg‐Eppendorf Hamburg Germany; ^7^ German Center for Cardiovascular Research (DZHK), Partner Site North Hamburg Germany; ^8^ Department of Cardiology, Angiology and Intensive Care Medicine, Benjamin Franklin Campus (CBF) German Heart Center of the Charité—Universitätsmedizin Berlin Berlin Germany; ^9^ German Centre for Cardiovascular Research (DZHK), Partner Site Berlin Berlin Germany; ^10^ Friede Springer Center of Cardiovascular Prevention at Charité – Universitätsmedizin Berlin Berlin Germany; ^11^ Comprehensive Heart Failure Center (CHFC) University Clinic Würzburg Würzburg Germany; ^12^ Medical Clinic 1 University Clinic Würzburg Würzburg Germany; ^13^ Department of Experimental Pharmacology and Toxicology University Medical Center Hamburg‐Eppendorf Hamburg Germany; ^14^ Institute for Experimental Cardiovascular Medicine University Heart Center Freiburg · Bad Krozingen, University of Freiburg Freiburg Germany; ^15^ Department of Congenital Heart Disease and Paediatric Cardiology University Heart Center Freiburg · Bad Krozingen, University of Freiburg Freiburg Germany; ^16^ Faculty of Medicine University of Freiburg Freiburg Germany; ^17^ German Cancer Research Center (DKFZ) Heidelberg Germany; ^18^ Department of Nephrology, Rheumatology, Clinical Immunology and Hypertension RWTH Aachen, Medical Faculty Aachen Germany; ^19^ Institute of Biomedical Engineering Karlsruhe Institute of Technology (KIT) Karlsruhe Germany; ^20^ Heart and Diabetes Center NRW University Hospital of the Ruhr‐University Bochum, Erich & Hanna Klessmann‐Institute Bad Oeynhausen Germany; ^21^ Institute of Experimental Cardiovascular Research and University Center of Cardiovascular Sciences, University Medical Center Hamburg Eppendorf Hamburg Germany; ^22^ Department of Physiology, Anatomy and Genetics (DPAG) University of Oxford Oxford UK; ^23^ Department of Congenital Heart Disease and Paediatric Cardiology University Hospital Schleswig‐Holstein Kiel Germany; ^24^ German Centre for Cardiovascular Research (DZHK) Partner Site North Kiel Germany; ^25^ Center for Biomedical Research and Translational Surgery Medical University of Vienna Vienna Austria; ^26^ Formerly Head of Livestock Biotechnology, School of Life Sciences, Senior Excellence Faculty Technical University of Munich Munich Germany; ^27^ Institute for Cardiovascular Physiology Goethe University Frankfurt Am Main Germany; ^28^ German Centre for Cardiovascular Research (DZHK) Partner Site Rhine‐Main Frankfurt Germany; ^29^ Institute of Cellular and Molecular Physiology Friedrich‐Alexander‐University (FAU) Erlangen‐Nürnberg Erlangen Germany; ^30^ Department of Cardiology Kerckhoff‐Clinic Bad Nauheim Germany; ^31^ Cardio‐Pulmonary Institute (CPI) Giessen Germany; ^32^ Cluster of Excellence “Multiscale Bioimaging: From Molecular Machines to Networks of Excitable Cells” (MBExC) University of Göttingen Göttingen Germany; ^33^ German Center for Neurodegenerative Diseases (DZNE) Göttingen Germany; ^34^ German Center for Child and Adolescent Health (DZKJ) Göttingen Germany; ^35^ Fraunhofer Institute for Translational Medicine and Pharmacology (ITMP) Göttingen Germany; ^36^ Cluster of Excellence “Centre for Integrative Biological Signalling Studies” (CIBSS) University of Freiburg Freiburg Germany

**Keywords:** animal models, donor tissue, human myocardium, stem cell models, vertebrate models

## Abstract

Model systems that mimic human cardiac structure and function are essential for the development of novel diagnostics and effective treatments for cardiovascular diseases. While non‐human vertebrate models, from zebrafish to pig, remain vital to cardiovascular research, the translatability of findings to human patients is often limited. Therefore, animal experiments should be supplemented with human model systems, including human induced pluripotent stem cell‐derived cells, 3D engineered constructs, and last but not least, native tissue preparations and isolated primary cardiomyocytes. However, while human myocardium remains the gold standard, human heart tissue – and particularly tissue from control hearts–remains scarce, and its use in research is generally restricted to settings where tissue has been excised from diseased or failing hearts. While it is in principle possible to use tissue from rejected non‐failing donor hearts that cannot be transplanted, legal hurdles (e.g., in Germany) can restrict the use of non‐transplanted donor organs in research. Given the challenges associated with accessing and using human tissue in biomedical research, an integrated strategy towards combining non‐human vertebrate models, in silico models, and human tissue‐derived models is recommended, enhancing the chances of successful research and development, and helping bridge the gap between preclinical and clinical research.

## Graphical abstract

This manuscript reviews models that can be used to study the physiology and pathophysiology of the heart, and describes some the limitaions and ethical concerns associated with the use of human tissue.

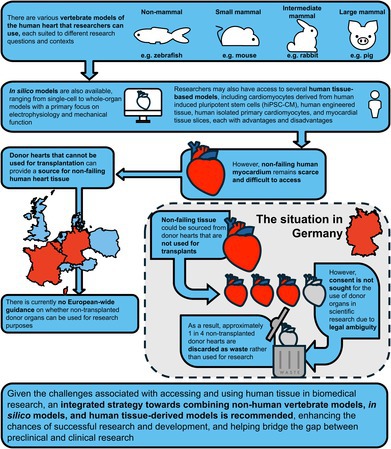

## 1 INTRODUCTION

The development of novel, affordable, and effective treatments for cardiovascular diseases greatly relies on the use of model systems that mimic human cardiac structure and function. While human heart tissue would be the best model for investigating human cardiovascular function and pathology, its limited availability (which is subject to ethical and legal restrictions) means that non‐human models play crucial roles in cardiovascular research and development. However, the extent to which cardiovascular diseases can be recapitulated ex vivo is limited, owing to complex aetiologies, the involvement of extra‐cardiac influences on the heart (e.g. circulating hormones, immune cells, and nervous system effects), and additional factors such as age, sex, and comorbidities, as well as the progressive nature of structural and functional pathological remodeling (which are all in turn affected by factors such as environmental cues, lifestyle, and medical treatments). This complexity cannot easily be mimicked in vitro, and it may not be well captured in non‐human vertebrate models either. As a result, the translatability of basic science findings to patients has remained limited, and the vast majority of candidate drugs that enter human testing fail, often due to a lack of efficacy or unexpected toxicity in spite of prior promising results in non‐human models (Austin, 2021; Barter et al., 2007; Harrison, 2016; Ineichen et al., 2024; Morehouse et al., 2007; Perry & Lawrence, 2017; Sun et al., 2022). Combinations of models that mimic human pathophysiological mechanisms more accurately are therefore needed to improve bio‐medical translation (Thomas, Desai, & Takahashi, 2022).

This paper is based on discussions at the 5th Translational Workshop of The German Cardiac Society (DGK) and the German Centre for Cardiovascular Research (DZHK) in Hamburg, Germany, September 2024, dedicated to *Models of Human Myocardium in Medical Research*. It reviews existing in vivo, ex vivo, in vitro, and in silico models, and discusses the utility and limitations of using human cells and tissue for clinically motivated research, with special consideration of the research landscape in Germany.

## 2 NON‐HUMAN VERTEBRATE MODELS OF THE HEART FOR CARDIAC RESEARCH

**FIGURE 1:**
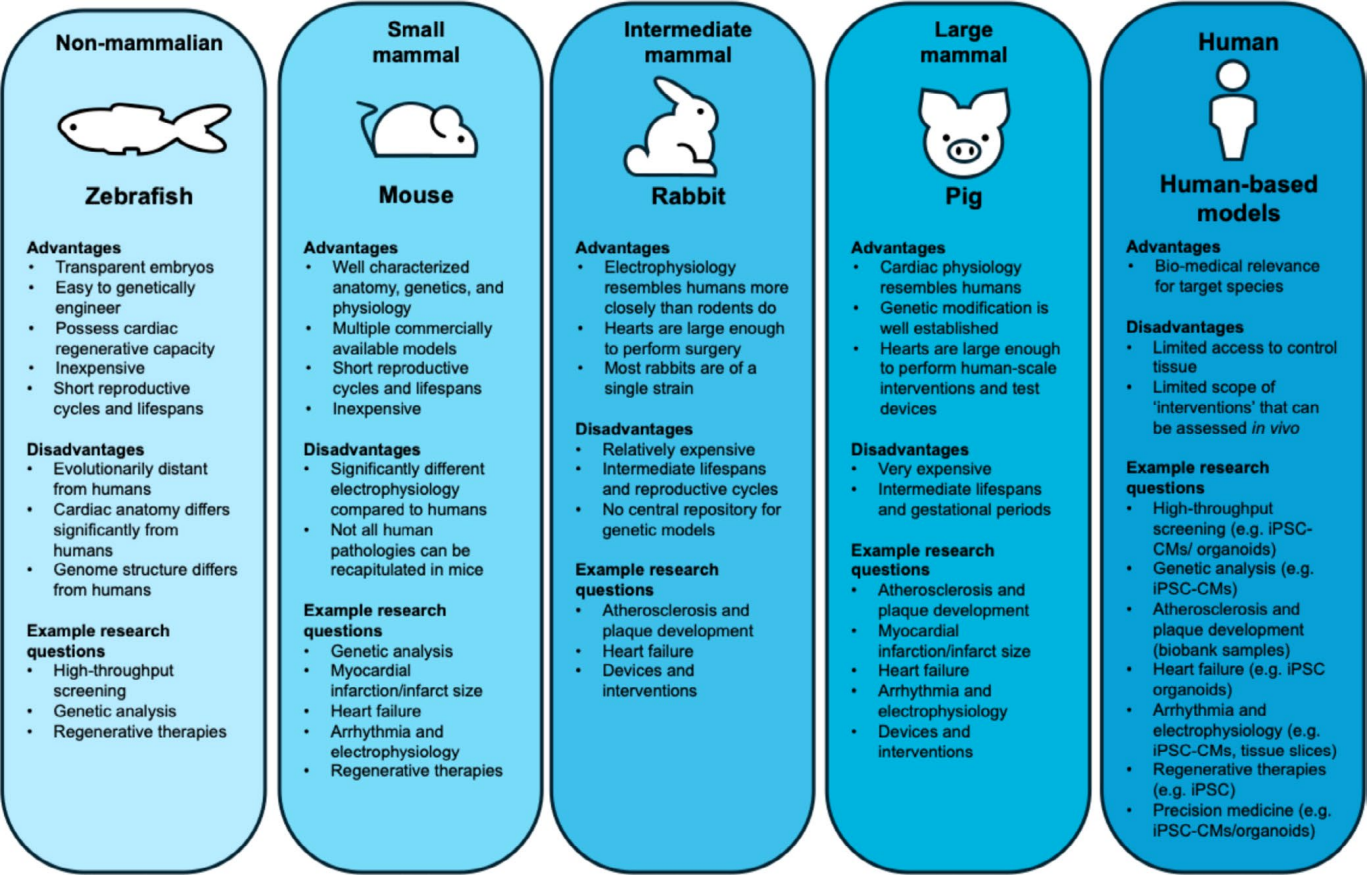
Advantages and disadvantages of various vertebrate models used in cardiovascular research (Tsang et al., 2016; van der Velden et al., 2022; Zaragoza et al., 2011).

Non‐human vertebrate models allow cardiovascular researchers to perform experiments that cannot be performed on humans, while facilitating the identification of novel therapeutic and treatment approaches, before these are tested in patients (Giacomotto & Ségalat, 2010; Odening et al., 2021). Vertebrate models can be used to mimic complex inter‐organ (e.g., metabolic, neurohormonal, or immunological) crosstalk with the heart, while allowing control of factors ranging from genetic background to aging, comorbidities, and treatments. Here, we review some of the more commonly used vertebrate models in cardiac research (see also Table 1). The advantages and disadvantages of each model, as well as examples of research questions for each, are shown in Figure 1 (Tsang et al., 2016; van der Velden et al., 2022; Zaragoza et al., 2011).

**TABLE 1:** Key parameters of selected vertebrate models commonly used in cardiovascular research (other commonly used models include invertebrates, chick embryos, rats, sheep, goats, non‐human primates, and historically also cats and dogs).

Parameter	Non‐mammalian, e.g. zebrafish	Small mammal, e.g. mouse	Intermediate mammal, e.g. rabbit	Large mammal, e.g. pig	Human
Body weight	0.5 g	20–45 g	1–5 kg	40–90 kg	50–100 kg
Heart weight	0.5 mg	0.15–0.2 g	5–10 g	145–450 g	150–480 g
Resting heart rate	110–170 bpm	450–800 bpm	140–280 bpm	70–120 bpm	60–100 bpm
Cardiac output	0.09–0.172 mL/min	13–15 mL/min	260–420 mL/min	2720–3620 mL/min	3340–9790 mL/min
Ejection fraction	40%–50%	65%–90%	55%–65%	50%–55%	55%–60%
Ventricular action potential duration	150–250 ms	80–180 ms	150–280 ms	180–300 ms	200–400 ms
Conduction velocity	4–10 mm/s	350–600 mm/s	300–500 mm/s	500–800 mm/s	500–900 mm/s
Reentry space factor^a^	0.42	0.34	0.69	1.43	1.5
Approximate cost per animal	€2.00–2.50/tank	€4–7	€250–600	€80–400+	‐
Husbandry notes	Swarm fish, density 4–5 animals per liter	Individually ventilated or standard cages	Larger pens	Large facilities or catheterisation laboratories, low volume but translational	‐
Anesthesia, and effects on cardiovascular endpoints	Tricaine: bradycardia, decreased heart rate and contractility, effects dose‐dependent	Isoflurane and opioids: decreased heart rate and blood pressure	Volatile or injectable anesthetics: altered haemodynamics, effects on heart rhythm	Isoflurane and similar: more frequent arrhythmias, infarct variability, effects on blood pressure effects	‐
Typical n per group in cardiovascular studies	30–100+ embryos	8–15	6–10	6–12	‐

*Note*: Color indicates good (green) or satisfactory (yellow) match to human cardiac parameters.

^a^ Calculated as maximum ventricular circumference divided by (conduction velocity × action potential duration).

### 2.1 Zebrafish: An example of non‐mammalian model systems

Despite being evolutionarily distant from primates, non‐mammalian animal models such as zebrafish and chick embryos (Rees et al., 2026; Wittig & Münsterberg, 2020) have been used to model several human cardiovascular disorders, including congenital heart defects and cardiomyopathies (Asnani & Peterson, 2014; MacRae & Peterson, 2015).

A unique advantage of zebrafish is that their embryos are transparent, allowing for in vivo optical measurements of cardiac structure and function. Additionally, their genome can be manipulated relatively easily, and they possess high regenerative capacities that may provide opportunities to identify novel targets for human cardiac repair, such as following ischaemic injury (Asnani & Peterson, 2014; Cesarovic et al., 2020). Zebrafish have a short breeding cycle and high fecundity, which permits rapid generation of models (Yang et al., 2024).

Crucially, zebrafish have hearts that share electrophysiological properties with human hearts, including conserved fundamentals of excitation–contraction coupling, similar action potential trajectories (both species have a pronounced plateau phase, for example), and comparable major inward and outward current systems, including sodium, calcium, and potassium currents (Vornanen & Hassinen, 2016). However, while useful for biochemical assays such as drug screening, zebrafish hearts differ from human hearts in a host of biophysical properties, including chamber anatomy, size, and haemodynamics, meaning that findings cannot easily be translated to humans.

In conclusion, zebrafish are well‐suited for live imaging, genetic and drug screening, as well as developmental and cardiac electrophysiological research. Yet, they differ substantially in size, structure, mechanical activity, and genetic make‐up from humans, which can complicate translation of findings (Figure 1).

### 2.2 Mice: The most widely used small mammalian model

Mice are the most commonly used animals in biomedical research (Hickman et al., 2017), due in part at least to the availability of standardized recombinatorial lines that allow a level of genetic manipulation unmatched in other vertebrate models (Gurumurthy & Kent Lloyd, 2019). Since the first report of a transgenic mouse (Gordon et al., 1980), the cumulative global production of transgenic founders has reached the millions (Hanahan et al., 2007). Other key advantages of the mouse as a model system are early sexual maturation, short gestational periods, and comparatively short lifespans, allowing for studies over multiple developmental stages of an individual, or even over generations. These features, along with their small size, comparatively low cost of maintenance, and the relative ease of surgical interventions, have contributed to the widespread use of mice in cardiovascular research (Hickman et al., 2017; Justice & Dhillon, 2016).

Given the wide range of ready‐to‐use models that are commercially available, mice are a convenient choice for experimental investigations. Murine anatomy, genetics, and physiology have been well studied, and reference values are well established. Furthermore, mouse models of a wide variety of diseases and cardiac conditions, including cardiomyopathies, heart failure, and developmental defects, have been established (Chowdhury et al., 2015; Lindsey et al., 2021; Rao et al., 2022; Salerno et al., 2023; Zaragoza et al., 2011). However, due to substantial differences in physiological cardiac function (such as heart rate, action potential shape and duration, calcium dynamics, etc.; Table 1), as well as in responses to pathological stimuli and therapeutic interventions–including resistance to the development of atrial fibrillation (Fu et al., 2022) and atherosclerosis (Emini Veseli et al., 2017)–the translatability of findings in mice to human patients remains limited. In addition, one of the most commonly used mouse strains (C57BL/6J) carries a loss‐of‐function mutation of the gene encoding the mitochondrial transhydrogenase, which makes it resistant to a number of common aetiologies of heart failure, such as pressure‐overload induced left or right heart failure, hypertrophic cardiomyopathy, and heart failure with a preserved ejection fraction, induced by high fat diet and the vasopressor N(ω)‐nitro‐L‐arginine methyl ester (L‐NAME) (Kohlhaas et al., 2024; Müller et al., 2022; Nickel et al., 2015; Pepin et al., 2025; Schiattarella et al., 2019). ‘Humanized’ mice, which are genetically modified to express functional human genes, have been proposed to extend the utility and translational potential of murine models (Rosshart et al., 1979). In addition, several central repositories for genetic mouse models now contain recently created wild‐derived mice that better represent human genetic background diversity (www.mmrrc.org; www.findmice.org (Donahue et al., 2012; Eppig et al., 2015)).

Thus, the mouse is a valuable model for both genetic and epigenetic manipulation, enabling thorough mechanistic studies of various disease conditions and developmental disorders. Limitations include differences in size, genetics, and electro‐mechanical function compared to humans (Figure 1).

### 2.3 Rabbits: The intermediate size mammalian model

Rabbits (commonly New Zealand white) have been dubbed “the largest of the small mammalian models” in cardiovascular research (Odening & Kohl, 2016). While rabbit hearts are 30 to 40 times smaller than human hearts by weight, they are large enough to allow use of scaled‐down instrumentation used in human interventions, including surgical and implantable devices (Pogwizd & Bers, 2008). Rabbit hearts resemble human hearts much more closely than mouse hearts in terms of cellular electrophysiology, including the shape of action potentials and arrhythmia wavelength, as well as their response to ischaemia and pharmacological interventions (Odening et al., 2020; Panfilov, 2006; Pogwizd & Bers, 2008). In contrast to mice and rats, rabbits also display a positive force–frequency relationship with calcium handling properties that are similar to humans (Endoh, 2004; Milani‐Nejad & Janssen, 2014). Furthermore, both electrophysiological and mechanical remodeling associated with aging and disease resemble key changes that occur in humans (Alpert et al., 1992; Cooper et al., 2012).

Disadvantages compared to murine models include the later sexual maturity, longer reproductive cycle, and longer lifespan of rabbits, making multi‐generational and aging studies more challenging. Rabbit housing and maintenance are also more costly. There are relatively few transgenic rabbit models and, in contrast to mice, these do not allow the easy “mix‐and‐match” recombination that systems such as the established Cre/LoxP or Flp/FRT lines offer for mice. Regrettably, there is no central repository for genetic rabbit models, leading to duplication of efforts in generating models, and at least occasionally to the loss of transgenic model lines maintained by individual labs (Hornyik et al., 2022). Encouragingly, however, genetic manipulation via point mutations and small insertions can work well, and new genetic techniques using TALEN or CRISPR/Cas9 have simplified the generation of transgenic mammals, including rabbits (Liu et al., 2018; Yang et al., 2019).

Overall, the rabbit is a valuable model for studying cardiac electrophysiology and mechanics in health and disease. Limitations include relatively high demands for time and resources, as well as the limited availability of transgenic models (compared to mice), as well as their somewhat unusual immune system (with one variable heavy chain and an unusually complex immunoglobulin A system) (Figure 1).

### 2.4 Pigs: A “human‐sized” mammalian model

Several large vertebrate models are used in cardiovascular research, of which non‐human primates have the closest phylogenetic relationship to humans. However, other large vertebrate models including goats, sheep, or pigs are also used.

Like humans, pigs are large, omnivorous, diurnal mammals, and the two species share relevant similarities in terms of anatomy, physiology, and biochemistry, with the pig's cardiovascular system being considered a good model for humans (Cesarovic et al., 2020). Pigs and humans have hearts of similar size and mass, and there is overlap in terms of heart rate, cardiac output, and ejection fraction (Table 1). Accordingly, pigs have been used as models for many cardiovascular diseases, including atherosclerosis, hypercholesterolemia, hypertrophic cardiomyopathy, as well as for the exploration of cardiac regeneration and cell therapy (Davis et al., 2014; Huang et al., 2017; Liu et al., 2021; Montag et al., 2018; Sridharan et al., 2023). Genetic modification is well established for the pig, including conditional gene targeting (Fischer & Schnieke, 2023) and blastocyst complementation (Barlabé et al., 2025). Examples of in vivo genome editing of the porcine heart have been published (Moretti et al., 2020; Rieblinger et al., 2021). In view of the high financial and logistic burdens, generation and maintenance of genetically modified pig models is conducted by few labs worldwide (Prather et al., 2013). Porcine‐expanded potential stem cells have also been differentiated into cardiomyocytes, which may provide a useful platform for preclinical testing of cardiac therapies (Gao et al., 2019; Rawat et al., 2023). Given the similarities in size and cardiac anatomy of pigs and humans, pigs are also used for developing and testing cardiovascular devices for diagnosis and intervention (Miller et al., 2016). In addition, research involving pigs also holds promise for xenotransplantation, including the potential to grow humanized hearts in pigs, although significant challenges remain (Cooper & Cozzi, 2024; Garry et al., 2025).

Pigs are comparatively slow to reach sexual maturity (5–6 months) and have long gestational periods (approximately 16 weeks) and lifespans (up to 20 years), making generational and aging studies difficult. In addition, while the possibility of using clinical equipment for research on pigs makes them a favored model for device development and procedure testing, the associated infrastructural demands, such as for operating theater facilities, raise the threshold for wider use.

Therefore, due to similarities between porcine and human hearts, pigs are a valuable translational model for research into pathogenesis, diagnostics, and drug‐ or device‐based therapy. Limitations include their long reproductive cycle and lifespan, often underestimated phylogenetic distance to humans, high costs, and infrastructural demands (Figure 1).

### 2.5 Challenges of using non‐human vertebrate models of the heart

Vertebrate models of the human heart are vital to cardiovascular research, particularly where experiments cannot be performed on humans. However, it is important that researchers consider interspecies differences that may affect the translatability of results, including mitochondrial function and dynamics (Alibrandi & Lionetti, 2025), ion currents (Horváth et al., 2020; Linz & Meyer, 2000), DNA methylation and gene expression (Pai et al., 2011), as well as cellular (Greiner et al., 2026) and myocardial architecture (Mulbjerg et al., 2025). In addition, researchers should be aware of other model‐specific differences, such as in seasonal (different for farm‐ and laboratory‐housed species) and circadian rhythms (especially for nocturnal animals, such as mice, which usually are studied at various points during their rest period, which involves substantial changes, e.g., in cardiomyocyte electrophysiology (Shen et al., 2007)), menstrual cycles (rabbits, for example, do not have a typical oestrous cycle but are induced ovulators, which must be taken into account when exploring sex differences (Giammarino et al., 2025)), and diet (as an example, standard murine chow is soy‐based and, as such, high in phytoestrogens, which affects hormone balance and cardiac function in particular in male mice (Stauffer, 2005)).

Regardless of the model chosen, mimicking clinical background therapies, which are one of the central determinants of translational validity in experimental cardiovascular research (van der Velden et al., 2022), is also a crucial consideration. Evaluating new interventions without taking standard‐of‐care treatments into account can lead to findings that are difficult to interpret or apply in real clinical settings.

The use of living organisms as models of human disease also requires carefully balancing potential benefits for human health that can be gained against the distress, pain, and/or suffering of animals involved. Scientific research is guided by the 3R principles, referring to “replacement” when possible, “reduction” of the number of animals used, and “refinement” of protocols, interventions, and upkeep to minimize animal discomfort while maximizing scientific insight. Implicit in this is a fourth R: responsibility on the part of the investigator for the experimental animal (Hubrecht & Carter, 2019; Lee et al., 2020; Tannenbaum & Bennett, 2015). Computational modeling and simulation can contribute to refined planning of animal experiments, to data integration, and to projection from animal‐based data for estimation of effects in humans (Morotti et al., 2021; Viceconti et al., 2021).

The use of non‐human vertebrate models in pharmaceutical screening suffers from several principal drawbacks. Both false‐positive (when therapies appear effective in animals but fail in human trials) and false‐negative observations (when therapies that would perform well in humans perform poorly in animal models and are abandoned prematurely) are common (Perry & Lawrence, 2017; van der Velden et al., 2022). This explains the desire for human models, from in vitro use of patient cells to exploration of tissue and organ explants, and on to clinical data mining approaches. The aim here is to improve the accuracy of testing, and to reduce the percentage of false‐negative and false‐positive conclusions in pre‐clinical development before human trials are commenced. Thus, human wet and dry experimental models are needed to bridge the gap between bench and bedside more effectively.

## 3 HUMAN MODELS FOR CARDIAC RESEARCH

### 3.1 Human induced pluripotent stem cells and human engineered heart tissue

Cardiomyocytes derived from human induced pluripotent stem cells (hiPSC‐CM) provide a sustainable source of cells that can be maintained in cell culture for months. hiPSC can be differentiated into several cardiomyocyte subtypes, including atrial, ventricular, and pacemaker‐like cells (Cyganek et al., 2018; Protze et al., 2017). These cells can be used for basic research in genetics, signaling, biophysics, or regeneration. Genome manipulation, such as introducing mutations, deletions, insertions, knockouts, or knock‐ins, enables the creation of disease models, investigation of the consequences of genome alterations and of genetic defect corrections, and the use of reporters and optogenetic actuators to study cell function (Bengel et al., 2021; Haertter et al., 2024; Kime et al., 2016; Stüdemann et al., 2022; Wang, McCain, et al., 2014).

The unique genetic relationship with their donors provides a potential for the exploration of genetic causes of cardiac disorders at a patient‐specific level (Cuello et al., 2021; Itzhaki et al., 2011; Moretti et al., 2010; Streckfuss‐Bömeke et al., 2017; Wu et al., 2024). This has supported therapy selection for individual patients (Prondzynski et al., 2019) and allowed re‐classification of genetic variants of hitherto unknown importance (Ma et al., 2018).

One possibility to improve the functional depth and robustness of readouts, the maturity of hiPSC‐CM, and the similarity to native heart muscle is to engineer 3D cardiac tissues and to incorporate other cell types such as fibroblasts, smooth muscle, endothelial or immune cells. These 3D models are not only increasingly being used as a platform for gene and cell therapy studies, drug screening or disease modeling (Borchert et al., 2017; Fomin et al., 2021; Greer‐Short et al., 2025; Hinson et al., 1979; Kyriakopoulou et al., 2023; Kyrychenko et al., 2017; Mannhardt et al., 2016; Saleem et al., 2020; Streckfuss‐Bömeke et al., 2017), but they are making their way into direct clinical use (Jebran et al., 2025). Combinations of multiple cell types in tissue‐engineered heart muscle emerge as a critical precondition for maturing hiPSC‐CM. These models allow for cell–cell interaction studies (Landau et al., 2024; Tiburcy et al., 2017), including research in genetically mixed models to determine the contribution of specific cell types to a phenotype or investigations into genome repair for restoration of cardiac function in disease (Long et al., 2018). Novel tissue constructs, such as assembloids composed of atrial, atrioventricular, and ventricular cardiomyocyte spheroids, allow for the study of complex disorders affecting heart rhythm (Li et al., 2024; Mallapaty, 2025). Combining multiple tissue‐engineered model systems furthermore opens the door to organ–organ interaction studies (Schneider et al., 2023).

Key limitations of hiPSC‐CM include their metabolic, electrophysiological, and structural immaturity, as the cells generally resemble embryonic or neonatal, rather than mature cardiomyocytes. Given this limitation, maturation strategies for iPSC‐CM are actively being explored on multiple fronts, including long‐term culture (Emanuelli et al., 2022), use of different culture media (Feyen et al., 2020), including protocols to foster metabolic maturation (Emanuelli et al., 2022; Feyen et al., 2020; Li et al., 2025; Rebs et al., 2025; Wickramasinghe et al., 2022), and mechanical or electrical stimulation (Hirt et al., 2014), incorporation into 3D heart organoids, or combinatorial approaches (Tan & Ye, 2018). Thus far, however, the most mature hiPSC‐CM remain phenotypically different from adult cardiomyocytes (Yang et al., 2023). Another challenge of working with hiPSC‐CM is managing variability that occurs from clone to clone, from differentiation to differentiation, between passages, and from laboratory to laboratory–even when nominally using the same cell lines and protocols (Thomas, Cunningham, et al., 2022). These limitations underscore the need for generally agreed‐upon quality assessment routines. Stem cell journals and societies have published relevant guidelines (Selfa Aspiroz et al., 2025) and adopted minimal standards that, for example, require the use of isogenic controls when studying the effects of genetic variants and validation of results in several cell lines, sufficient numbers of replicates, and independent differentiation batches.

### 3.2 Human isolated primary cardiomyocytes and non‐cardiomyocytes

Unlike hiPSC‐CM, primary cardiomyocytes are directly isolated from human myocardial tissue, obtained from different regions of the heart. These cells retain core characteristics of mature cardiomyocytes, including their structural, functional, and epigenetic profile (Zhou et al., 2022), while keeping patient‐specific phenotypes (e.g., related to age, sex, comorbidities, medical treatments, and the progressive remodeling associated with cardiac diseases). These native phenotypes make findings more directly translatable. Isolated cardiomyocytes allow for a high degree of experimental control and provide a comparatively inexpensive, convenient pathophysiologically relevant model for evaluating cellular behavior and morphology (Oh et al., 2019; Pitoulis et al., 2020), as well as ion channel function, contraction and electrophysiological characteristics (Odening et al., 2021). Human primary cardiomyocytes have traditionally been used both in basic and translational research, particularly in signaling studies and drug efficacy/toxicity testing (Ahmad et al., 2019; Grammatika Pavlidou et al., 2023; Molina et al., 2018; Odening et al., 2021). More recently, culturing human primary cardiomyocytes without overt dedifferentiation changes has enabled genetic manipulation, which can in turn be used to study the effects of genome alterations, as proof of concept for gene therapies, to investigate the consequences of tachypacing over time, to analyze biomarker release and function, or to utilize genetically encoded biosensors to study cellular function for example (Aceituno et al., 2024; Beneke & Molina, 2022; Berisha et al., 2021; Grammatika Pavlidou et al., 2023; Pabel et al., 2022).

However, the functional viability of isolated cells is limited to a relatively short period, typically no more than 1 day to 1 week (Aceituno et al., 2024; Beneke & Molina, 2022), before they progressively lose their structural and functional integrity (Banyasz et al., 2008; Beneke & Molina, 2022; Greiner et al., 2022; Guo et al., 2018; Mitcheson et al., 1996; Seidel et al., 2019; Zhou et al., 2022). Cold preservation of mouse hearts and human isolated myocytes can extend the time during which they can be used in functional research without major structural, functional, or transcriptional effects (Aceituno et al., 2024; Pfeilschifter et al., 2025). This time window can also be extended by isolating cells on consecutive days from living cardiac tissue slices (discussed in the next section (Greiner et al., 2022)), as live tissue slices can be maintained for several weeks (Brandenburger et al., 2012). Whether or not even longer‐term cryopreservation of cells, with subsequent recovery of key live cell function, is indeed plausible remains to be elucidated (Wang et al., 2026). Although multiple cell types (e.g., cardiomyocytes, fibroblasts, neurons) can be obtained during the isolation process, so that cell–cell interaction studies are possible, cellular models cannot be used to study long‐term therapeutic interventions or to replicate in vivo complexity and systemic physiological connections.

### 3.3 Myocardial tissue slices

Myocardial tissue slices are living sections of human heart tissue that are usually cut to a thickness of 250–350 μm and kept in culture while mimicking certain physiological conditions. They retain key aspects of the structure (e.g., matrix/cell integration), function (e.g., electrical activity and contractions), and heterocellular signaling of native tissue (Brandenburger et al., 2012; Fischer et al., 2019; Pitoulis et al., 2020; Wang, Terrar, et al., 2014; Watson et al., 2019). Remarkably, myocardial tissue slices can maintain electrophysiological behavior that is close to that of the same tissue‐block surface before cutting (as shown in slices from rabbit hearts (Wang, Terrar, et al., 2014)). They also maintain contractility, gene expression profiles and the response to important pharmacological or hormonal stimuli for up to 3 months (Fischer et al., 2019; Klumm et al., 2022; van der Geest et al., 2025). Similar to hiPSC‐CM and 3D models, myocardial slices can be used to explore disease mechanisms and can be matched with patient data (Abu‐Khousa et al., 2020). They can also be used to evaluate or identify the therapeutic effects of drugs (Amesz et al., 2024; Krammer et al., 2025), and for the study of cardiac contractility modulation (Bierhuizen et al., 2025) and cardiac toxicity screening (Shi et al., 2023). This model can also be used to separate human myocardium from the extracardiac neural innervation that may otherwise confound results in vivo, providing a tissue model with controlled conditions. Direct effects on the myocardium can thus be studied in high detail and with high fidelity. Furthermore, due to the extended viability in culture (Fischer et al., 2019; Habeler et al., 2009), the study of long‐term effects, such as of cardiac tissue remodeling, becomes possible. The analysis of the culture medium offers another opportunity, as released metabolic products, cytokines or enzymes, as well as nutrients can be quantified (Baron et al., 2024).

Limitations of myocardial slices include the tissue damage that occurs during slicing, which directly affects near‐surface cell layers. Furthermore, given the transmurally varying alignment of cardiac cells relative to the cutting plane, the functional variability from slice to slice, even if cut from the same tissue block, can be significant. Furthermore, although permitting a high level of control, for example, over the diastolic load or the composition of the culture medium, the local biophysical (e.g., stress/strain conditions, electrical source‐sink relations) and biochemical environments (e.g., nutrient accessibility, hormonal signaling, oxygen delivery) differ from in vivo conditions (Baron et al., 2024). Slices are also disconnected from the circulation and nervous system, so that the exploration of inter‐organ crosstalk, of effects of the immune system, and of response to systemic signals is restricted.

Despite these limitations, myocardial tissue slices represent one of the closest approximations to native human myocardium available in vitro. For myocardial tissue slices to realize their full translational potential, coordinated efforts towards protocol harmonization, inter‐laboratory reproducibility assessment, and consensus definition of minimal reporting standards will be essential (Quinn et al., 2011).

### 3.4 In silico models

Computational models of the human heart span from single‐cell to organ‐in‐chest levels, with a primary focus on electrophysiology and mechanical function. Mechanistic in silico models are versatile and scalable, allowing researchers to explore various physiological and pathological scenarios in a well‐controlled and cost‐effective way, and without the use of animal or human samples (Lai et al., 2025; Niederer et al., 2019; Trayanova et al., 2024; Yamamoto et al., 2026; Zhang et al., 2026). Validated models can predict emergent behavior on different biological levels of integration, to optimize data interpretation, diagnosis, and treatment. In direct iteration with wet‐lab research, they can serve as the start (hypothesis‐generating) and end (data interpretation) of integrated knowledge development (Loewe et al., 2025), though start and end are arbitrary designations in what is probably more akin to a spiral of knowledge development (Quinn & Kohl, 2013). In combination with artificial intelligence‐based data assessment, mechanistic in silico models are becoming increasingly predictive (Corral‐Acero et al., 2020; Roney et al., 2022) and are beginning to directly aid personalized cardiac treatment (Lai et al., 2025; Sakata et al., 2024; Sakata et al., 2026; Shade et al., 2021; Waight et al., 2025).

Regulatory acceptance of in silico data is increasing. The Comprehensive in vitro Proarrhythmia Assay (CiPA) initiative, aimed to develop a novel, mechanistic, and model‐informed assessment of the pro‐arrhythmic potential of new drugs, including the use of in silico computer modeling to calculate pro‐arrhythmic risk scores (as well as 2D and 3D hiPSC‐CM models), is an example of this development, as it involves regulatory agencies such as the FDA and EMA as stakeholders (Li et al., 2020; Vicente et al., 2018). In April 2025, for example, the FDA announced a phasing out of mandatory animal testing for some drug types, to be replaced with computational models (among other sources of data) (Food and Drug Administration, 2026). The same month, the National Institutes of Health announced a plan to prioritize human‐based research technologies, including computational models that “simulate complex human systems, disease pathways, and drug interactions” (National Institutes of Health, 2026). The use of in silico models to assess cardiovascular safety is therefore increasingly embedded within regulatory science and drug development (Ridder et al., 2020). As computational approaches are poised to inform high‐consequence decisions, their credibility must be established within structured risk‐based frameworks such as V&V40 (Verification & Validation 40) (U.S. Department of Health and Human Services, 2018), and aligned with the emerging principles of Good Simulation Practices (GSP) (Viceconti & Emili, 2024). These frameworks emphasize rigorous verification (ensuring that the computational implementation correctly represents underlying mathematical models), validation (demonstrating that model outputs are consistent with the relevant biological or clinical reality for the defined context of use), and uncertainty quantification (characterizing parametric, structural, and methodological uncertainties and their impact on decision‐relevant outputs).

Limitations of in silico models include the fact that their utility depends on the quality and extent of input data, both for mechanistic and probabilistic models. And while computational approaches can help translate insight across species, validation using human data remains paramount.

## 4 AVAILABILITY OF HUMAN TISSUE FOR CARDIAC RESEARCH

Human cardiac tissue availability is a bottleneck for basic research. Myocardial tissue cannot be obtained by voluntary live‐donation, and while biopsy material or samples from surgical interventions may be collected for certain clinically diagnostic purposes, access to control tissue from non‐diseased hearts is not an option, except for post‐mortem or (in some countries) non‐transplanted donor hearts. The use of human heart tissue for functional and molecular research is therefore generally restricted to settings where it has been excised for medical reasons, such as atrial tissue resected for cardiac bypass surgery, ventricular tissue excised during aortic valve replacement or for ventricular assist device implantation, during septal myectomy in patients with obstructive hypertrophic cardiomyopathy or aortic stenosis, or whole hearts excised from transplant recipients. In all these scenarios, the excised tissue is from diseased hearts, so that “control experiments” compare one medical condition with another (e.g., atrial tissue from patients with atrial fibrillation can be compared to tissue from patients in sinus rhythm–but both groups include patients with cardiac conditions that necessitated cardiac surgery). There is therefore an obvious unmet need for non‐failing human heart tissue that can be used as a reference for control investigations. Post‐mortem organ donation of non‐failing heart tissue for research and teaching is possible, but the suitability of tissue for functional studies is determined by the time taken to access it, as delays of more than a few minutes pose major limitations for studies relying on metabolically competent organs and cells. A realistic source of live non‐failing control myocardium would be donor hearts that, for unforeseen reasons, cannot be used for their primary medical purpose–transplantation–but this is not always possible (Iaizzo, 2016).

### 4.1 Use of non‐failing human heart tissue for scientific research: Challenges and opportunities

**FIGURE 2:**
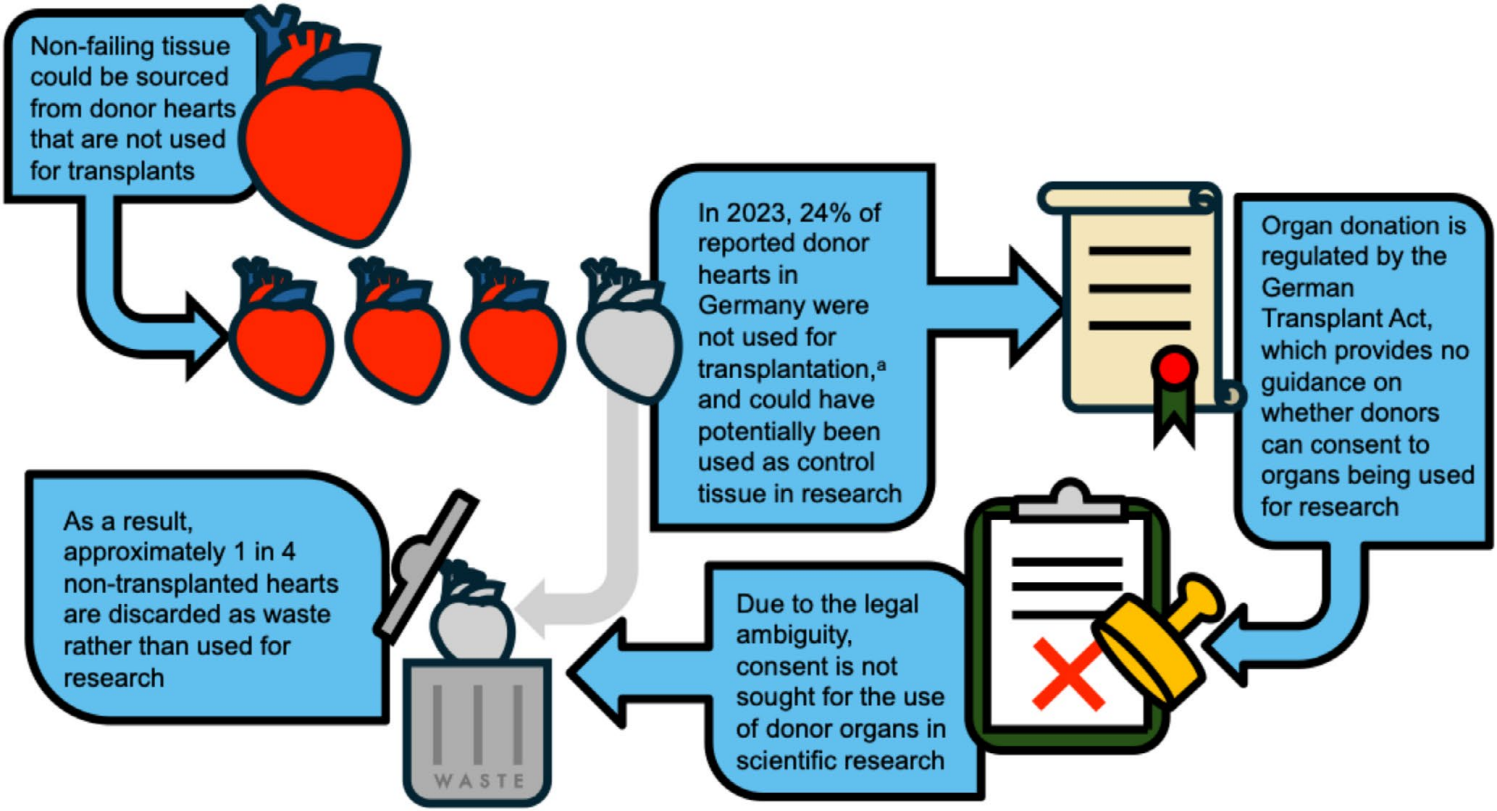
Why non‐failing hearts are not used as control tissue in scientific research in Germany. ^a^Eurotransplant Statistics Report Library 2024 (Library, 2023).

Access to non‐failing control myocardium in the form of rejected donor hearts is problematic in Germany. While there is a shortage of organ donors in Germany and elsewhere anyway (Bunnik, 2023; Eurotransplant, 2024; IRODaT, 2024; Library, 2023; Pick & Krug, 2024; Statista, 2024), not all available organs can be transplanted. For example, 96 of the 399 hearts (24%) that would have been available for transplantation in 2023 were not used (Figure 2). Whatever the reasons for non‐transplantation, those donor organs may not be utilized for research in Germany. This means that about one in four donor hearts end up as biological waste, despite the fact that a single donor heart could provide enough human cells and control tissue to support dozens, if not hundreds of research projects utilizing in vitro models, such as live tissue slices, isolated cells, or cultures. This waste occurs because German transplantation legislation does not include (and hence, does not permit) the use of donor organs for any other than medical purposes. Documentation of donor consent is focused exclusively on tissue and organ use for direct therapeutic purposes, and options in case of non‐transplantation, including the potential use of donated tissue in research or education, are not included in these forms. This contrasts with the UK, for example, where the donor or the relatives/nominated representatives are consulted, using a clearly structured consent/authorisation form. This is completed by the National Health Service (NHS) staff during consultation and it includes specific tick‐box questions covering secondary (non‐medical) uses, such as for research, training, or quality assurance. The overall rate of consent for research use of organs found to be unsuitable for transplantation is very high, at 93% for the UK as a whole (Mumford, 2022). The lack of a similar structured approach to consent for secondary uses in Germany, by contrast, means that it is difficult (if not impossible) to even approximate the willingness of patients to consent to their tissue being used for research purposes.

Additional confusion arises around the legal “ownership” of an organ. Once it has been assigned to a recipient by Eurotransplant, for example, ‘ownership’ is formally transferred to the recipient. But–there is no guidance on whose consent (the donor's or recipient's) would be required for non‐medical use if an organ is eventually not transplanted. Due to this legal ambiguity, donor organs which cannot be transplanted are currently discarded (Figure 2).

**FIGURE 3:**
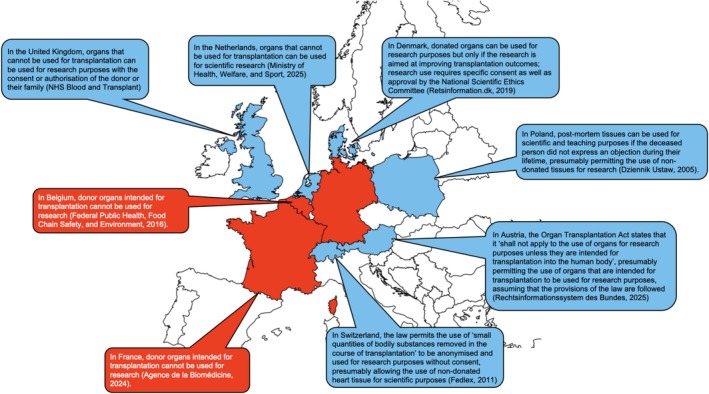
Laws and guidelines relating to the use of non‐transplanted donor hearts for research in selected European countries (Dossier de presse, 2024; Fedlex. Federal Act, 2011; Health Food Chain Safety Environment, 2016; NHS Blood and Transplant, 2026; Rechtsinformationssystem des Bundes, 2026; Retsinformation.dk, 2026; Scientific Research, 2026; Ustaw, 2026).

This situation is part of the patchwork of regulations relating to organ donation across Europe, where the use of donor tissue is generally determined on a national level (Figure 3), and which may add an additional layer of legal complexity around questions of ownership and consent, as organs are regularly shared between countries during the process of donor–recipient matching. This situation contrasts with the USA, where the use of human tissue in research is governed by federal, state, and local laws, regulations, and policies, including the Department of Health and Human Service's “Common Rule” and the Health Insurance Portability and Accountability Act (Bledsoe & Grizzle, 2013; Grizzle, 2019). In practice, human heart tissue can be obtained for scientific research via tissue banks and networks like the National Cancer Institute‐funded Cooperative Human Tissue Network (https://chtn.cancer.gov/), Texas Children's Hospital's Heart Center Tissue Bank (https://www.texaschildrens.org/research/find‐laboratory/heart‐center‐tissue‐bank), and the International Institute for the Advancement of Medicine (https://iiam.org/researchers/human‐tissue‐for‐research/). These provide non‐transplantable organs and tissues (including cardiac tissue) for use in medical research on a national scale.

A key question raised in this context is whether or not it is ethically acceptable (e.g., consistent with donor intentions) for tissue that cannot be used for the originally intended purpose (clinical application) to be used for secondary objectives such as medical research, training, or quality control. It may be assumed that organ donors are altruistic, as they intend their donation to benefit others. Whether this includes the indirect benefits of scientific research into cardiac diseases cannot be assessed retrospectively. However, the high proportion of donors (or their families) who consent to their tissues being used for research purposes when given the option (93% in the UK, 89% in the Netherlands, for example (Lutomski & Manders, 2024; Mumford, 2022)) suggests that donor intentions may be broader than simply supporting direct medical use. This is also evident from the very high proportion of patients who are willing to donate (diseased) tissue for use in scientific research, once it has been excised for medical reasons (Fitzpatrick et al., 2009; Jack & Womack, 2003). This means that, in view of the uniquely relevant roles of control tissue for research, an alternative question should be posed–namely whether it is ethically acceptable for tissue that cannot be transplanted to be simply discarded, instead of being used in secondary applications.

The legislative situation in Germany regarding organ donation seems unlikely to improve soon (Bundestag, 2020; Kappler & Kohl, 2023; Rehsmann, 2023; Welle, 2020), as is evident from the response of the German government (Deutscher Bundestag 20, 2023a) to a formal parliamentary enquiry on this very topic (Deutscher Bundestag 20, 2023b) in 2023. Since the use of organs or tissue, originally intended for transplantation, for research, teaching, or quality control is unaddressed (i.e., neither permitted nor prohibited per se) in Germany, it would seem to be prudent to consider changing the informed consent process, in collaboration with organizations conducting it for organ donation, perhaps to a model similar to that of the UK. This may involve the creation of shared standard operating procedures to ensure that organs which cannot be used for transplantation do not go to waste, as well as the establishment of a coordinated national (or European) procurement network to handle, process, and share tissue for research, to prevent the current waste of uniquely valuable control tissue.

## 5 CONCLUSIONS

There are multiple model systems for human cardiovascular research, including animal tissue, iPSC‐derived cells, primary human tissue and cells, and computational models. All these models have inherent benefits and limitations, related to how well they represent specific aspects of human cardiac structure and function, as well as reproducibility, amenability to conducting controlled investigations, cost, and access, to name but a few. While pronounced inter‐species differences can be bridged (at least in part) using theoretical models, human heart tissue remains the gold standard for clinically relevant cardiac research. The scarcity of human heart tissue, in particular from non‐failing control hearts, is compounded by the fact that donor tissue that cannot be used for its intended primary medical purpose is not yet systematically available for academic research, training, and quality control, with divergent regulations in individual European countries (Figure 3). The UK provides a good example of how consent for primary and secondary uses of organs can be effectively obtained, with very high rates of consent. Conversely, legal ambiguities, such as in Germany, can hinder the use of precious donor tissue for the advancement of medical insight. This calls, on the one hand, for careful combination of multiple model systems leveraging their respective strengths, and on the other hand, for a broader discussion with all stakeholders about the handling of donated tissue for medical progress in the present (i.e., treatment) and future (i.e., research).

## AUTHOR CONTRIBUTIONS

**Katrin Streckfuss‐Bömeke:** Conceptualization. **Laura C. Zelarayán:** Conceptualization. **Renate B. Schnabel:** Conceptualization. **Nicolle Kränkel:** Conceptualization. **Christoph Maack:** Conceptualization. **Thomas Eschenhagen:** Conceptualization. **Hannah E. Kappler:** Conceptualization. **Ursula Klingmüller:** Conceptualization. **Rafael Kramann:** Conceptualization. **Axel Loewe:** Conceptualization. **Hendrik Milting:** Conceptualization. **Cristina E. Molina:** Conceptualization. **Daniela Panáková:** Conceptualization. **Bruno K. Podesser:** Conceptualization. **Angelika Schnieke:** Conceptualization. **Katrin Schröder:** Conceptualization. **Thomas Seidel:** Conceptualization. **Samuel Sossalla:** Conceptualization. **Callum Zgierski‐Johnston:** Conceptualization. **Wolfram‐Hubertus Zimmermann:** Conceptualization. **Eva A. Rog‐Zielinska:** Conceptualization. **Peter Kohl:** Conceptualization.

## FUNDING INFORMATION

This paper is based on discussions at the 5th DGK/DZHK Translational Workshop “Models of human myocardium in functional medical research: opportunities and challenges” held in Hamburg, Germany, 25 September 2024, organized by the Commission for Experimental Cardiovascular Medicine of the DGK. The workshop was financially supported by DGK, Novartis AG, and Boehringer Ingelheim International GmbH.

## CONFLICT OF INTEREST STATEMENT

Katrin Streckfuss‐Bömeke: speakers' honoraria from Novartis. Christoph Maack: speakers/consulting honoraria from Astra Zeneca, Boehringer Ingelheim, Bristol Myers Squibb, Cytokinetics, Lilly, Novo Nordisk. Thomas Eschenhagen: advisor and member of board of directors of Dinabios AG. Rafael Kramann: founder, equity holder, and advisor Sequantrix GmbH. Bruno K. Podesser: advisor to HeartbeatBio GmbH. Thomas Seidel: equity holder in InVitroSys GmbH. Samuel Sossalla: speakers/consulting honoraria from Astra Zeneca, Novartis, Berlin‐Chemie, Daiichi Sankyo, Bristol Myers Squibb, Pfizer, Boehringer Ingelheim, Lilly. Wolfram‐Hubertus Zimmermann: founder of, equity holder in, and advisor to Repairon GmbH and myriamed GmbH. All other authors have no conflicts to declare.

## ETHICS STATEMENT

There was no ethics approval required for this study.

## Data Availability

Data sharing not applicable to this article, as no datasets were generated or analyzed.

## References

[phy270845-bib-0001] Abu‐Khousa, M. , Fiegle, D. J. , Sommer, S. T. , Minabari, G. , Milting, H. , & Heim, C. (2020). The degree of t‐system Remodeling predicts negative force‐frequency relationship and prolonged relaxation time in failing human myocardium. Frontiers in Physiology, 11, 182. 10.3389/fphys.2020.00182 32231589 PMC7083140

[phy270845-bib-0002] Aceituno, C. , Revuelta, D. , Jiménez‐Sábado, V. , Ginel, A. , Molina, C. E. , & Hove‐Madsen, L. (2024). Impact of overnight storage of human atrial myocytes on intracellular calcium homeostasis and electrophysiological utility. Biomolecules, 14, 1415. 10.3390/biom14111415 39595591 PMC11591567

[phy270845-bib-0003] Ahmad, S. , Tirilomis, P. , Pabel, S. , Dybkova, N. , Hartmann, N. , & Molina, C. E. (2019). The functional consequences of sodium channel NaV1.8 in human left ventricular hypertrophy. ESC. Heart Fail, 6, 154–163. 10.1002/ehf2.12378 PMC6352890 30378291

[phy270845-bib-0004] Alibrandi, L. , & Lionetti, V. (2025). Interspecies differences in mitochondria: Implications for cardiac and vascular translational research. Vascular Pharmacology, 159, 107476. 10.1016/j.vph.2025.107476 40037508

[phy270845-bib-0005] Alpert, N. R. , Hasenfuss, G. , Mulieri, L. A. , Blanchard, E. M. , Leavitt, B. J. , & Ittleman, F. (1992). The reorganization of the human and rabbit heart in response to haemodynamic overload. European Heart Journal, 13, 9–16. 10.1093/eurheartj/13.suppl_D.9 1396867

[phy270845-bib-0006] Amesz, J. H. , Langmuur, S. J. J. , Zhang, L. , Manintveld, O. C. , Schinkel, A. F. L. , & de Jong, P. L. (2024). Biomechanical response of ultrathin slices of hypertrophic cardiomyopathy tissue to myosin modulator mavacamten. Biomedicine and Pharmacotherapy, 170, 116036. 10.1016/j.biopha.2023.116036 38134635

[phy270845-bib-0007] Asnani, A. , & Peterson, R. T. (2014). The zebrafish as a tool to identify novel therapies for human cardiovascular disease. Disease Models & Mechanisms, 7, 763–767. 10.1242/dmm.016170 24973746 PMC4073266

[phy270845-bib-0008] Austin, C. P. (2021). Opportunities and challenges in translational science. Clinical and Translational Science, 14, 1629–1647. 10.1111/cts.13055 33982407 PMC8504824

[phy270845-bib-0009] Banyasz, T. , Lozinskiy, I. , Payne, C. E. , Edelmann, S. , Norton, B. , & Chen, B. (2008). Transformation of adult rat cardiac myocytes in primary culture. Experimental Physiology, 93, 370–382. 10.1113/expphysiol.2007.040659 18156167

[phy270845-bib-0010] Barlabé, P. , Aranguren, X. L. , & Coppiello, G. (2025). Blastocyst complementation: Current progress and future directions in xenogeneic organogenesis. Stem Cell Res Ther, 16, 321. 10.1186/s13287-025-04426-y 40551151 PMC12186422

[phy270845-bib-0011] Baron, V. , Sommer, S. T. , Fiegle, D. J. , Pfeuffer, A.‐K. M. , Peyronnet, R. , & Volk, T. (2024). Effects of electro‐mechanical uncouplers, hormonal stimulation and pacing rate on the stability and function of cultured rabbit myocardial slices. Front Bioeng. Biotech, 12, 1363538. 10.3389/fbioe.2024.1363538 PMC11026719 38646013

[phy270845-bib-0012] Barter, P. J. , Caulfield, M. , Eriksson, M. , Grundy, S. M. , Kastelein, J. J. P. , & Komajda, M. (2007). Effects of Torcetrapib in patients at high risk for coronary events. New England Journal of Medicine, 357, 2109–2122. 10.1056/nejmoa0706628 17984165

[phy270845-bib-0013] Beneke, K. , & Molina, C. E. (2022). Live cell imaging of cyclic nucleotides in human Cardiomyocytes. Methods in Molecular Biology, 2483, 195–204. 10.1007/978-1-0716-2245-2_12 35286677

[phy270845-bib-0014] Bengel, P. , Dybkova, N. , Tirilomis, P. , Ahmad, S. , Hartmann, N. A. , & Mohamed, B. (2021). Detrimental proarrhythmogenic interaction of Ca^2+^/calmodulin‐dependent protein kinase II and NaV1.8 in heart failure. Nature Communications, 12, 6586. 10.1038/s41467-021-26690-1 PMC8593192 34782600

[phy270845-bib-0015] Berisha, F. , Götz, K. R. , Wegener, J. W. , Brandenburg, S. , Subramanian, H. , & Molina, C. E. (2021). CAMP imaging at ryanodine receptors reveals β2‐Adrenoceptor driven arrhythmias. Circulation Research, 129, 81–94. 10.1161/CIRCRESAHA.120.318234 33902292

[phy270845-bib-0016] Bierhuizen, M. F. A. , Amesz, J. H. , Langmuur, S. J. J. , Lam, B. , Knops, P. , & Veen, K. M. (2025). Acute biomechanical effects of cardiac contractility modulation in living myocardial slices from end‐stage heart failure patients. Bioengineering, 12, 174. 10.3390/bioengineering12020174 40001693 PMC11851609

[phy270845-bib-0017] Bledsoe, M. J. , & Grizzle, W. E. (2013). Use of human specimens in research: The evolving United States regulatory, policy, and scientific landscape. Diagnostic Histopathology, 19, 322–330. 10.1016/j.mpdhp.2013.06.015 24639889 PMC3954467

[phy270845-bib-0018] Borchert, T. , Hübscher, D. , Guessoum, C. I. , Lam, T.‐D. D. , Ghadri, J. R. , & Schellinger, I. N. (2017). Catecholamine‐dependent β‐adrenergic Signaling in a pluripotent stem cell model of Takotsubo cardiomyopathy. Journal of the American College of Cardiology, 70, 975–991. 10.1016/j.jacc.2017.06.061 28818208

[phy270845-bib-0019] Brandenburger, M. , Wenzel, J. , Bogdan, R. , Richardt, D. , Nguemo, F. , & Reppel, M. (2012). Organotypic slice culture from human adult ventricular myocardium. Cardiovascular Research, 93, 50–59. 10.1093/cvr/cvr259 21972180

[phy270845-bib-0020] Bundestag, D. (2020). Organspenden: Mehrheit für die Entscheidungslösung. https://www.bundestag.de/dokumente/textarchiv/2020/kw03‐de‐transplantationsgesetz‐674682

[phy270845-bib-0021] Bunnik, E. M. (2023). Ethics of allocation of donor organs. Current Opinion in Organ Transplantation, 28, 192–196. 10.1097/MOT.0000000000001058 36787240 PMC10155689

[phy270845-bib-0022] Cesarovic, N. , Lipski, M. , Falk, V. , & Emmert, M. Y. (2020). Animals in cardiovascular research. European Heart Journal, 41, 200–203. 10.1093/eurheartj/ehz933 31909425

[phy270845-bib-0023] Chowdhury, R. , Ashraf, H. , Melanson, M. , Tanada, Y. , Nguyen, M. , & Silberbach, M. (2015). Mouse model of human congenital heart disease: Progressive atrioventricular block induced by a heterozygous Nkx2‐5 homeodomain missense mutation. Circulation. Arrhythmia and Electrophysiology, 8, 1255–1264. 10.1161/CIRCEP.115.002720 26226998 PMC4618020

[phy270845-bib-0024] Cooper, D. K. C. , & Cozzi, E. (2024). Clinical pig heart xenotransplantation—Where do we go from here? Transplant International, 37, 12592. 10.3389/ti.2024.12592 38371908 PMC10869462

[phy270845-bib-0025] Cooper, L. L. , Odening, K. E. , Hwang, M.‐S. , Chaves, L. , Schofield, L. , & Taylor, C. A. (2012). Electromechanical and structural alterations in the aging rabbit heart and aorta. American Journal of Physiology. Heart and Circulatory Physiology, 302, H1625–H1635. 10.1152/ajpheart.00960.2011 22307668 PMC4747897

[phy270845-bib-0026] Corral‐Acero, J. , Margara, F. , Marciniak, M. , Rodero, C. , Loncaric, F. , & Feng, Y. (2020). The ‘digital twin’ to enable the vision of precision cardiology. European Heart Journal, 41, 4556–4564. 10.1093/eurheartj/ehaa159 32128588 PMC7774470

[phy270845-bib-0027] Cuello, F. , Knaust, A. E. , Saleem, U. , Loos, M. , Raabe, J. , & Mosqueira, D. (2021). Impairment of the ER/mitochondria compartment in human cardiomyocytes with PLN p.Arg14del mutation. EMBO Molecular Medicine, 13, EMMM202013074. 10.15252/emmm.202013074 PMC8185541 33998164

[phy270845-bib-0028] Cyganek, L. , Tiburcy, M. , Sekeres, K. , Gerstenberg, K. , Bohnenberger, H. , & Lenz, C. (2018). Deep phenotyping of human induced pluripotent stem cell‐derived atrial and ventricular cardiomyocytes. JCI Insight, 3, e99941. 10.1172/jci.insight.99941 29925689 PMC6124434

[phy270845-bib-0029] Davis, B. T. , Wang, X. J. , Rohret, J. A. , Struzynski, J. T. , Merricks, E. P. , & Bellinger, D. A. (2014). Targeted disruption of LDLR causes hypercholesterolemia and atherosclerosis in Yucatan miniature pigs. PLoS One, 9, e93457. 10.1371/journal.pone.0093457 24691380 PMC3972179

[phy270845-bib-0030] Deutscher Bundestag 20 . (2023a). Wahlperiode. Antwort der Bundesregierung auf die Kleine Anfrage der Fraktion der CDU/CSU–Drucksache 20/7480–Nutzung postmortaler Spender‐Organe zu Forschungszwecken. Drucksache. https://dserver.bundestag.de/btd/20/077/2007731.pdf

[phy270845-bib-0031] Deutscher Bundestag 20 . (2023b). Wahlperiode. Kleine Anfrage der Fraktion der CDU/CSU Nutzung postmortaler Spender‐Organe zu Forschungszwecken. Drucksache. https://dserver.bundestag.de/btd/20/074/2007480.pdf

[phy270845-bib-0032] Donahue, L. R. , Hrabe de Angelis, M. , Hagn, M. , Franklin, C. , Lloyd, K. K. , Magnuson, T. , & McKerlie, C. (2012). Centralized mouse repositories. Mammalian Genome, 23, 559–571. 10.1007/s00335-012-9420-4 22945696 PMC3709583

[phy270845-bib-0033] Dossier de presse . (2024). Agence de la biomédicine. Baromètre d'opinion. https://back.agence‐biomedecine.fr/uploads/Dossier_de_presse_complet_en_PDF_6598766daa.pdf

[phy270845-bib-0034] Emanuelli, G. , Zoccarato, A. , Reumiller, C. M. , Papadopoulos, A. , Chong, M. , & Rebs, S. (2022). A roadmap for the characterization of energy metabolism in human cardiomyocytes derived from induced pluripotent stem cells. Journal of Molecular and Cellular Cardiology, 164, 136–147. 10.1016/j.yjmcc.2021.12.001 34923199

[phy270845-bib-0035] Emini Veseli, B. , Perrotta, P. , De Meyer, G. R. A. , Roth, L. , Van der Donckt, C. , & Martinet, W. (2017). Animal models of atherosclerosis. European Journal of Pharmacology, 816, 3–13. 10.1016/j.ejphar.2017.05.010 28483459

[phy270845-bib-0036] Endoh, M. (2004). Force–frequency relationship in intact mammalian ventricular myocardium: Physiological and pathophysiological relevance. European Journal of Pharmacology, 500, 73–86. 10.1016/j.ejphar.2004.07.013 15464022

[phy270845-bib-0037] Eppig, J. T. , Motenko, H. , Richardson, J. E. , Richards‐Smith, B. , & Smith, C. L. (2015). The international mouse strain resource (IMSR): Cataloging worldwide mouse and ES cell line resources. Mammalian Genome, 26, 448–455. 10.1007/s00335-015-9600-0 26373861 PMC4602064

[phy270845-bib-0038] Eurotransplant . (2024). Deutschland. https://www.eurotransplant.org/region/deutschland/

[phy270845-bib-0039] Fedlex. Federal Act . (2011). On research involving human beings. SR, 810(30), 2011. https://www.fedlex.admin.ch/eli/cc/2013/617/en

[phy270845-bib-0040] Feyen, D. A. M. , McKeithan, W. L. , Bruyneel, A. A. N. , Spiering, S. , Hörmann, L. , & Ulmer, B. (2020). Metabolic maturation media improve physiological function of human iPSC‐derived Cardiomyocytes. Cell Reports, 32, 107925. 10.1016/j.celrep.2020.107925 32697997 PMC7437654

[phy270845-bib-0041] Fischer, C. , Milting, H. , Fein, E. , Reiser, E. , Lu, K. , & Seidel, T. (2019). Long‐term functional and structural preservation of precision‐cut human myocardium under continuous electromechanical stimulation in vitro. Nature Communications, 10, 117. 10.1038/s41467-018-08003-1 PMC6328583 30631059

[phy270845-bib-0042] Fischer, K. , & Schnieke, A. (2023). How genome editing changed the world of large animal research. Frontiers in Genome Editing, 5, 1272687. 10.3389/fgeed.2023.1272687 37886655 PMC10598601

[phy270845-bib-0043] Fitzpatrick, P. E. , McKenzie, K. D. , Beasley, A. , & Sheehan, J. D. (2009). Patients attending tertiary referral urology clinics: Willingness to participate in tissue banking. BJU International, 104, 209–213. 10.1111/j.1464-410X.2009.08666.x 19493259

[phy270845-bib-0044] Fomin, A. , Gärtner, A. , Cyganek, L. , Tiburcy, M. , Tuleta, I. , & Wellers, L. (2021). Truncated titin proteins and titin haploinsufficiency are targets for functional recovery in human cardiomyopathy due to *TTN* mutations. Science Translational Medicine, 13, eabd3079. 10.1126/scitranslmed.abd3079 34731013

[phy270845-bib-0045] Food and Drug Administration . (2026). FDA Announces Plan to Phase Out Animal Testing Requirement for Monoclonal Antibodies and Other Drugs 2025. https://www.fda.gov/news‐events/press‐announcements/fda‐announces‐plan‐phase‐out‐animal‐testing‐requirement‐monoclonal‐antibodies‐and‐other‐drugs

[phy270845-bib-0046] Fu, F. , Pietropaolo, M. , Cui, L. , Pandit, S. , Li, W. , & Tarnavski, O. (2022). Lack of authentic atrial fibrillation in commonly used murine atrial fibrillation models. PLoS One, 17, e0256512. 10.1371/journal.pone.0256512 34995278 PMC8741011

[phy270845-bib-0047] Gao, X. , Nowak‐Imialek, M. , Chen, X. , Chen, D. , Herrmann, D. , & Ruan, D. (2019). Establishment of porcine and human expanded potential stem cells. Nature Cell Biology, 21, 687–699. 10.1038/s41556-019-0333-2 31160711 PMC7035105

[phy270845-bib-0048] Garry, D. J. , Garry, M. G. , Nakauchi, H. , Masaki, H. , Sachs, D. H. , & Weiner, J. I. (2025). Allogeneic, xenogeneic, and exogenic hearts for transplantation. Methodist DeBakey Cardiovascular Journal, 21, 92–99. doi:10.14797/mdcvj.1590 40384731 PMC12082467

[phy270845-bib-0049] Giacomotto, J. , & Ségalat, L. (2010). High‐throughput screening and small animal models, where are we? British Journal of Pharmacology, 160, 204–216. 10.1111/j.1476-5381.2010.00725.x 20423335 PMC2874843

[phy270845-bib-0050] Giammarino, L. , Matas, L. , Alerni, N. , Horváth, A. , Vashanthakumar, V. , & Nimani, S. (2025). Sex and sex hormonal regulation of the atrial inward rectifier potassium current (IK1): Insights into potential pro‐arrhythmic mechanisms. Cardiovascular Research, 121, 1215–1227. 10.1093/cvr/cvaf074 40272446 PMC12310280

[phy270845-bib-0051] Gordon, J. W. , Scangos, G. A. , Plotkin, D. J. , Barbosa, J. A. , & Ruddle, F. H. (1980). Genetic transformation of mouse embryos by microinjection of purified DNA. Proceedings of the National Academy of Sciences, 77, 7380–7384. 10.1073/pnas.77.12.7380 PMC350507 6261253

[phy270845-bib-0052] Grammatika Pavlidou, N. , Dobrev, S. , Beneke, K. , Reinhardt, F. , Pecha, S. , & Jacquet, E. (2023). Phosphodiesterase 8 governs cAMP/PKA‐dependent reduction of L‐type calcium current in human atrial fibrillation: A novel arrhythmogenic mechanism. European Heart Journal, 44, 2483–2494. 10.1093/eurheartj/ehad086 36810794 PMC10344654

[phy270845-bib-0053] Greer‐Short, A. , Greenwood, A. , Leon, E. C. , Qureshi, T. N. , von Kraut, K. , & Wong, J. (2025). AAV9‐mediated MYBPC3 gene therapy with optimized expression cassette enhances cardiac function and survival in MYBPC3 cardiomyopathy models. Nature Communications, 16, 2196. 10.1038/s41467-025-57481-7 PMC11880196 40038304

[phy270845-bib-0054] Greiner, J. , Schiatti, T. , Kaltenbacher, W. , Dente, M. , Semenjakin, A. , & Kok, T. (2022). Consecutive‐day ventricular and atrial Cardiomyocyte isolations from the same heart: Shifting the Cost–benefit balance of cardiac primary cell research. Cells, 11, 11. 10.3390/cells11020233 PMC8773758 35053351

[phy270845-bib-0055] Greiner, J. , Sonak, F. , Jones, W. D. , Madl, J. , Ryeng, K. A. , & Efimov, I. R. (2026). Architecture of the cardiac transverse‐axial tubular system across different mammalian species. BioRxiv, 2026.01.10.698376. 10.64898/2026.01.10.698376

[phy270845-bib-0056] Grizzle, W. E. (2019). Issues in the use of human tissues to support precision medicine. Journal of Health Care for the Poor and Underserved, 30, 66–78. 10.1353/hpu.2019.0117 31735720 PMC7985924

[phy270845-bib-0057] Guo, G. R. , Chen, L. , Rao, M. , Chen, K. , Song, J. P. , & Hu, S. S. (2018). A modified method for isolation of human cardiomyocytes to model cardiac diseases. Journal of Translational Medicine, 16, 288. 10.1186/s12967-018-1649-6 30348184 PMC6198433

[phy270845-bib-0058] Gurumurthy, C. B. , & Kent Lloyd, K. C. (2019). Generating mouse models for biomedical research: Technological advances. Disease Models & Mechanisms, 12, dmm029462. 10.1242/dmm.029462 30626588 PMC6361157

[phy270845-bib-0059] Habeler, W. , Peschanski, M. , & Monville, C. (2009). Organotypic heart slices for cell transplantation and physiological studies. Organogenesis, 5, 62–66. 10.4161/org.5.2.9091 19794901 PMC2710527

[phy270845-bib-0060] Haertter, D. , Hauke, L. , Driehorst, T. , Nishi, K. , Zimmermann, W.‐H. , & Schmidt, C. F. (2024). Sarcomere dynamic instability and stochastic heterogeneity drive robust cardiomyocyte contraction. BioRxiv, 2024.05.28.596183. 10.1101/2024.05.28.596183 PMC13574337 42734122

[phy270845-bib-0061] Hanahan, D. , Wagner, E. F. , & Palmiter, R. D. (2007). The origins of oncomice: A history of the first transgenic mice genetically engineered to develop cancer. Genes & Development, 21, 2258–2270. 10.1101/gad.1583307 17875663

[phy270845-bib-0062] Harrison, R. K. (2016). Phase II and phase III failures: 2013–2015. Nature Reviews. Drug Discovery, 15, 817–818. 10.1038/nrd.2016.184 27811931

[phy270845-bib-0063] Health Food Chain Safety Environment . (2016). Federal Public Health Food Chain Safety and environment. Organ donation and donation of body to science. Federal Public Health Food Chain Safety and Environment. https://www.health.belgium.be/en/organ‐donation‐and‐donation‐body‐science

[phy270845-bib-0064] Hickman, D. L. , Johnson, J. , Vemulapalli, T. H. , Crisler, J. R. , & Shepherd, R. (2017). Commonly Used Animal Models. Principles of Animal Research for Graduate and Undergraduate Students (pp. 117–175). Elsevier Inc. 10.1016/B978-0-12-802151-4.00007-4

[phy270845-bib-0065] Hinson, J. T. , Chopra, A. , Nafissi, N. , Polacheck, W. J. , Benson, C. C. , & Swist, S. (1979). Titin mutations in iPS cells define sarcomere insufficiency as a cause of dilated cardiomyopathy. Science, 2015(349), 982–986. 10.1126/science.aaa5458 PMC4618316 26315439

[phy270845-bib-0066] Hirt, M. N. , Boeddinghaus, J. , Mitchell, A. , Schaaf, S. , Börnchen, C. , & Müller, C. (2014). Functional improvement and maturation of rat and human engineered heart tissue by chronic electrical stimulation. Journal of Molecular and Cellular Cardiology, 74, 151–161. 10.1016/j.yjmcc.2014.05.009 24852842

[phy270845-bib-0067] Hornyik, T. , Rieder, M. , Castiglione, A. , Major, P. , Baczko, I. , & Brunner, M. (2022). Transgenic rabbit models for cardiac disease research. British Journal of Pharmacology, 179, 938–957. 10.1111/bph.15484 33822374

[phy270845-bib-0068] Horváth, B. , Hézső, T. , Szentandrássy, N. , Kistamás, K. , Árpádffy‐Lovas, T. , & Varga, R. (2020). Late sodium current in human, canine and Guinea pig ventricular myocardium. Journal of Molecular and Cellular Cardiology, 139, 14–23. 10.1016/j.yjmcc.2019.12.015 31958464

[phy270845-bib-0069] Huang, L. , Hua, Z. , Xiao, H. , Cheng, Y. , Xu, K. , & Gao, Q. (2017). CRISPR/Cas9‐mediated ApoE−/−and LDLR−/−double gene knockout in pigs elevates serum LDL‐C and TC levels. Oncotarget, 8, 37751–37760. doi:10.18632/oncotarget.17154 28465483 PMC5514946

[phy270845-bib-0070] Hubrecht, R. C. , & Carter, E. (2019). The 3Rs and humane experimental technique: Implementing change. Animals, 9, 754. 10.3390/ani9100754 31575048 PMC6826930

[phy270845-bib-0071] Iaizzo, P. A. (2016). The visible heart® project and free‐access website “atlas of human cardiac anatomy”. Europace, 18, 163–172. 10.1093/europace/euw359 28011844

[phy270845-bib-0072] Ineichen, B. V. , Furrer, E. , Grüninger, S. L. , Zürrer, W. E. , & Macleod, M. R. (2024). Analysis of animal‐to‐human translation shows that only 5% of animal‐tested therapeutic interventions obtain regulatory approval for human applications. PLoS Biology, 22, e3002667. 10.1371/journal.pbio.3002667 38870090 PMC11175415

[phy270845-bib-0073] IRODaT . (2024). Germany 2024. https://www.irodat.org/?p=database&c=DE#data

[phy270845-bib-0074] Itzhaki, I. , Maizels, L. , Huber, I. , Zwi‐Dantsis, L. , Caspi, O. , & Winterstern, A. (2011). Modelling the long QT syndrome with induced pluripotent stem cells. Nature, 471, 225–229. 10.1038/nature09747 21240260

[phy270845-bib-0075] Jack, A. L. , & Womack, C. (2003). Why surgical patients do not donate tissue for commercial research: Review of records. BMJ, 327, 262. 10.1136/bmj.327.7409.262 12896938 PMC167155

[phy270845-bib-0076] Jebran, A. F. , Seidler, T. , Tiburcy, M. , Daskalaki, M. , Kutschka, I. , & Fujita, B. (2025). Engineered heart muscle allografts for heart repair in primates and humans. Nature, 639, 503–511. 10.1038/s41586-024-08463-0 39880949 PMC11903342

[phy270845-bib-0077] Justice, M. J. , & Dhillon, P. (2016). Using the mouse to model human disease: Increasing validity and reproducibility. Disease Models & Mechanisms, 9, 101–103. 10.1242/dmm.024547 26839397 PMC4770152

[phy270845-bib-0078] Kappler, H. E. , & Kohl, P. (2023). Wissenschaftliche Nutzung von nicht transplantiertem Humangewebe. CardioNews. https://app.cardionews.de/Politik‐Gesellschaft/Wissenschaftliche‐Nutzung‐von‐nicht‐transplantiertem‐Humangewebe‐442919.html

[phy270845-bib-0079] Kime, C. , Mandegar, M. A. , Srivastava, D. , Yamanaka, S. , Conklin, B. R. , & Rand, T. A. (2016). Efficient CRISPR/Cas9‐based genome engineering in human pluripotent stem cells. Current Protocols in Human Genetics, 88, 2141–21423. 10.1002/0471142905.hg2104s88 PMC4726454 26724721

[phy270845-bib-0080] Klumm, M. J. , Heim, C. , Fiegle, D. J. , Weyand, M. , Volk, T. , & Seidel, T. (2022). Long‐term cultivation of human atrial myocardium. Frontiers in Physiology, 13, 839139. 10.3389/fphys.2022.839139 35283779 PMC8905341

[phy270845-bib-0081] Kohlhaas, M. , Sequeira, V. , Parikh, S. , Dietl, A. , Richter, O. , & Bay, J. (2024). Mitochondrial reactive oxygen species cause arrhythmias in hypertrophic cardiomyopathy. BioRxiv, 2024.10.02.616214. 10.1101/2024.10.02.616214

[phy270845-bib-0082] Krammer, T. , Baier, M. J. , Hegner, P. , Zschiedrich, T. , Lukas, D. , & Wolf, M. (2025). Cardioprotective effects of semaglutide on isolated human ventricular myocardium. European Journal of Heart Failure, 27, 1315–1325. 10.1002/ejhf.3644 40107718 PMC12370581

[phy270845-bib-0083] Kyriakopoulou, E. , Versteeg, D. , de Ruiter, H. , Perini, I. , Seibertz, F. , & Döring, Y. (2023). Therapeutic efficacy of AAV‐mediated restoration of PKP2 in arrhythmogenic cardiomyopathy. Nature Cardiovascular Research, 2, 1262–1276. 10.1038/s44161-023-00378-9 PMC11041734 38665939

[phy270845-bib-0084] Kyrychenko, V. , Kyrychenko, S. , Tiburcy, M. , Shelton, J. M. , Long, C. , & Schneider, J. W. (2017). Functional correction of dystrophin actin binding domain mutations by genome editing. JCI Insight, 2, e95918. 10.1172/jci.insight.95918 28931764 PMC5621913

[phy270845-bib-0085] Lai, C. , Yin, M. , Kholmovski, E. G. , Sani, M. M. , Gilotra, N. A. , & Chrispin, J. (2025). Predicting sudden cardiac death in patients with sarcoidosis using a multimodal artificial intelligence model. JACC Clin Electrophysiol, S2405‐500X(25)00829‐1. 10.1016/j.jacep.2025.10.009 41348081

[phy270845-bib-0086] Landau, S. , Zhao, Y. , Hamidzada, H. , Kent, G. M. , Okhovatian, S. , & Lu, R. X. Z. (2024). Primitive macrophages enable long‐term vascularization of human heart‐on‐a‐chip platforms. Cell Stem Cell, 31, 1222–1238. 10.1016/j.stem.2024.05.011 38908380 PMC11297673

[phy270845-bib-0087] Lee, K. H. , Lee, D. W. , & Kang, B. C. (2020). The ‘R’ principles in laboratory animal experiments. Laboratory Animal Research, 36, 45. 10.1186/s42826-020-00078-6 33298163 PMC7724623

[phy270845-bib-0088] Li, J. , Wiesinger, A. , Fokkert, L. , Bakker, P. , de Vries, D. K. , & Tijsen, A. J. (2024). Modeling the atrioventricular conduction axis using human pluripotent stem cell‐derived cardiac assembloids. Cell Stem Cell, 31, 1667–1684. 10.1016/j.stem.2024.08.008 39260368 PMC11546832

[phy270845-bib-0089] Li, W. , Luo, X. , Strano, A. , Arun, S. , Gamm, O. , & Poetsch, M. S. (2025). Comprehensive promotion of iPSC‐CM maturation by integrating metabolic medium with nanopatterning and electrostimulation. Nature Communications, 16, 2785. 10.1038/s41467-025-58044-6 PMC11928738 40118846

[phy270845-bib-0090] Li, Z. , Mirams, G. R. , Yoshinaga, T. , Ridder, B. J. , Han, X. , & Chen, J. E. (2020). General principles for the validation of Proarrhythmia risk prediction models: An extension of the CiPA in Silico strategy. Clinical Pharmacology and Therapeutics, 107, 102–111. 10.1002/cpt.1647 31709525 PMC6977398

[phy270845-bib-0091] Library, E. S. R. (2023). Heart donation, deceased donors in 2023, by country, by allocation phase.

[phy270845-bib-0092] Lindsey, M. L. , Brunt, K. R. , Kirk, J. A. , Kleinbongard, P. , Calvert, J. W. , & de Castro Brás, L. E. (2021). Guidelines for in vivo mouse models of myocardial infarction. American Journal of Physiology. Heart and Circulatory Physiology, 321, H1056–H1073. 10.1152/ajpheart.00459.2021 34623181 PMC8834230

[phy270845-bib-0093] Linz, K. W. , & Meyer, R. (2000). Profile and kinetics of L‐type calcium current during the cardiac ventricular action potential compared in Guinea‐pigs, rats and rabbits. Pflügers Archiv, 439, 588–599. 10.1007/s004249900212 10764219

[phy270845-bib-0094] Liu, S. , Li, K. , Florencio, L. W. , Tang, L. , Heallen, T. R. , & Leach, J. P. (2021). Gene therapy knockdown of hippo signaling induces cardiomyocyte renewal in pigs after myocardial infarction. Science Translational Medicine, 13, eabd6892. 10.1126/scitranslmed.abd6892 34193613 PMC9476348

[phy270845-bib-0095] Liu, Z. , Chen, M. , Chen, S. , Deng, J. , Song, Y. , & Lai, L. (2018). Highly efficient RNA‐guided base editing in rabbit. Nature Communications, 9, 2717. 10.1038/s41467-018-05232-2 PMC6045575 30006570

[phy270845-bib-0096] Loewe, A. , Hunter, P. J. , & Kohl, P. (2025). Computational modelling of biological systems now and then: Revisiting tools and visions from the beginning of the century. Philosophical Transactions of the Royal Society A: Mathematical, Physical and Engineering Sciences, 383, 20230384. 10.1098/rsta.2023.0384 PMC12105733 40336283

[phy270845-bib-0097] Long, C. , Li, H. , Tiburcy, M. , Rodriguez‐Caycedo, C. , Kyrychenko, V. , & Zhou, H. (2018). Correction of diverse muscular dystrophy mutations in human engineered heart muscle by single‐site genome editing. Science Advances, 4, eaap9004. 10.1126/sciadv.aap9004 29404407 PMC5796795

[phy270845-bib-0098] Lutomski, J. E. , & Manders, P. (2024). From opt‐out to opt‐in consent for secondary use of medical data and residual biomaterial: An evaluation using the RE‐AIM framework. PLoS One, 19, e0299430. 10.1371/journal.pone.0299430 38547214 PMC10977758

[phy270845-bib-0099] Ma, N. , Zhang, J. Z. , Itzhaki, I. , Zhang, S. L. , Chen, H. , & Haddad, F. (2018). Determining the pathogenicity of a genomic variant of uncertain significance using CRISPR/Cas9 and human‐induced pluripotent stem cells. Circulation, 138, 2666–2681. 10.1161/CIRCULATIONAHA.117.032273 29914921 PMC6298866

[phy270845-bib-0100] MacRae, C. A. , & Peterson, R. T. (2015). Zebrafish as tools for drug discovery. Nature Reviews. Drug Discovery, 14, 721–731. 10.1038/nrd4627 26361349

[phy270845-bib-0101] Mallapaty, S. (2025). Mini hearts, lungs and livers made in lab now grow their own blood vessels. Nature, 643, 892. 10.1038/d41586-025-02183-9 40646149

[phy270845-bib-0102] Mannhardt, I. , Breckwoldt, K. , Letuffe‐Brenière, D. , Schaaf, S. , Schulz, H. , & Neuber, C. (2016). Human engineered heart tissue: Analysis of contractile force. Stem Cell Reports, 7, 29–42. 10.1016/j.stemcr.2016.04.011 27211213 PMC4944531

[phy270845-bib-0103] Milani‐Nejad, N. , & Janssen, P. M. L. (2014). Small and large animal models in cardiac contraction research: Advantages and disadvantages. Pharmacology & Therapeutics, 141, 235–249. 10.1016/j.pharmthera.2013.10.007 24140081 PMC3947198

[phy270845-bib-0104] Miller, J. R. , Henn, M. C. , Lancaster, T. S. , Lawrance, C. P. , Schuessler, R. B. , & Shepard, M. (2016). Pulmonary valve replacement with small intestine submucosa‐extracellular matrix in a porcine model. World Journal for Pediatric and Congenital Heart Surgery, 7, 475–483. 10.1177/2150135116651113 27358303 PMC6959127

[phy270845-bib-0105] Mitcheson, J. S. , Hancox, J. C. , & Levi, A. J. (1996). Action potentials, ion channel currents and transverse tubule density in adult rabbit ventricular myocytes maintained for 6 days in cell culture. Pflügers Archiv, 431, 814–827. 10.1007/s004240050073 8927497

[phy270845-bib-0106] Molina, C. E. , Abu‐Taha, I. H. , Wang, Q. , Roselló‐Díez, E. , Kamler, M. , & Nattel, S. (2018). Profibrotic, electrical, and calcium‐handling remodeling of the atria in heart failure patients with and without atrial fibrillation. Frontiers in Physiology, 9, 1383. 10.3389/fphys.2018.01383 30356673 PMC6189336

[phy270845-bib-0107] Montag, J. , Petersen, B. , Flögel, A. K. , Becker, E. , Lucas‐Hahn, A. , & Cost, G. J. (2018). Successful knock‐in of hypertrophic cardiomyopathy‐mutation R723G into the MYH7 gene mimics HCM pathology in pigs. Scientific Reports, 8, 4786. 10.1038/s41598-018-22936-z 29555974 PMC5859159

[phy270845-bib-0108] Morehouse, L. A. , Sugarman, E. D. , Bourassa, P. A. , Sand, T. M. , Zimetti, F. , & Gao, F. (2007). Inhibition of CETP activity by torcetrapib reduces susceptibility to diet‐induced atherosclerosis in New Zealand white rabbits. Journal of Lipid Research, 48, 1263–1272. 10.1194/jlr.M600332-JLR200 17325387

[phy270845-bib-0109] Moretti, A. , Bellin, M. , Welling, A. , Jung, C. B. , Lam, J. T. , & Bott‐Flügel, L. (2010). Patient‐specific induced pluripotent stem‐cell models for Long‐QT syndrome. New England Journal of Medicine, 363, 1397–1409. 10.1056/nejmoa0908679 20660394

[phy270845-bib-0110] Moretti, A. , Fonteyne, L. , Giesert, F. , Hoppmann, P. , Meier, A. B. , & Bozoglu, T. (2020). Somatic gene editing ameliorates skeletal and cardiac muscle failure in pig and human models of Duchenne muscular dystrophy. Nature Medicine, 26, 207–214. 10.1038/s41591-019-0738-2 PMC7212064 31988462

[phy270845-bib-0111] Morotti, S. , Liu, C. , Hegyi, B. , Ni, H. , Fogli Iseppe, A. , & Wang, L. (2021). Quantitative cross‐species translators of cardiac myocyte electrophysiology: Model training, experimental validation, and applications. Science Advances, 7, eabg0927. 10.1126/sciadv.abg0927 34788089 PMC8598003

[phy270845-bib-0112] Mulbjerg, H. , Ringgaard, S. , & Agger, P. (2025). Diffusion tensor imaging reveals myocardial architectural differences between porcine and primate hearts with potential implications for cardiac xenotransplantation. Scientific Reports, 15, 28696. 10.1038/s41598-025-14368-3 40770390 PMC12329012

[phy270845-bib-0113] Müller, M. , Bischof, C. , Kapries, T. , Wollnitza, S. , Liechty, C. , & Geißen, S. (2022). Right heart failure in mice upon pressure overload is promoted by mitochondrial oxidative stress. Basic to Translational Science, 7, 658–677. 10.1016/j.jacbts.2022.02.018 35958691 PMC9357563

[phy270845-bib-0114] Mumford, L. N. H. S. (2022). Blood & transplant research, innovation and novel technologies advisory group research consent/authorisation rates.

[phy270845-bib-0115] National Institutes of Health . (2026). NIH to prioritize human‐based research technologies 2025. https://www.nih.gov/news‐events/news‐releases/nih‐prioritize‐human‐based‐research‐technologies

[phy270845-bib-0116] NHS Blood and Transplant . (2026). Understanding consent for organ donation. https://www.organdonation.nhs.uk/about‐organ‐donation/consent/

[phy270845-bib-0117] Nickel, A. G. , von Hardenberg, A. , Hohl, M. , Löffler, J. R. , Kohlhaas, M. , & Becker, J. (2015). Reversal of mitochondrial transhydrogenase causes oxidative stress in heart failure. Cell Metabolism, 22, 472–484. 10.1016/j.cmet.2015.07.008 26256392

[phy270845-bib-0118] Niederer, S. A. , Lumens, J. , & Trayanova, N. A. (2019). Computational models in cardiology. Nature Reviews. Cardiology, 16, 100–111. 10.1038/s41569-018-0104-y 30361497 PMC6556062

[phy270845-bib-0119] Odening, K. E. , Baczko, I. , & Brunner, M. (2020). Animals in cardiovascular research: Important role of rabbit models in cardiac electrophysiology. European Heart Journal, 41, 2036. 10.1093/eurheartj/ehaa251 32413901

[phy270845-bib-0120] Odening, K. E. , Gomez, A.‐M. , Dobrev, D. , Fabritz, L. , Heinzel, F. R. , & Mangoni, M. E. (2021). ESC working group on cardiac cellular electrophysiology position paper: Relevance, opportunities, and limitations of experimental models for cardiac electrophysiology research. EP Europace, 23, 1795–1814. 10.1093/europace/euab142 PMC11636574 34313298

[phy270845-bib-0121] Odening, K. E. , & Kohl, P. (2016). Follow the white rabbit: Experimental and computational models of the rabbit heart provide insights into cardiac (patho‐) physiology. Progress in Biophysics and Molecular Biology, 121, 75–76. 10.1016/j.pbiomolbio.2016.06.002 27315763

[phy270845-bib-0122] Oh, J. G. , Kho, C. , Hajjar, R. J. , & Ishikawa, K. (2019). Experimental models of cardiac physiology and pathology. Heart Failure Reviews, 24, 601–615. 10.1007/s10741-019-09769-2 30666533 PMC6561792

[phy270845-bib-0123] Pabel, S. , Knierim, M. , Stehle, T. , Alebrand, F. , Paulus, M. , & Sieme, M. (2022). Effects of atrial fibrillation on the human ventricle. Circulation Research, 130, 994–1010. 10.1161/CIRCRESAHA.121.319718 35193397 PMC8963444

[phy270845-bib-0124] Pai, A. A. , Bell, J. T. , Marioni, J. C. , Pritchard, J. K. , & Gilad, Y. (2011). A genome‐wide study of DNA methylation patterns and gene expression levels in multiple human and chimpanzee tissues. PLoS Genetics, 7, e1001316. 10.1371/journal.pgen.1001316 21383968 PMC3044686

[phy270845-bib-0125] Panfilov, A. V. (2006). Is heart size a factor in ventricular fibrillation? Or how close are rabbit and human hearts? Heart Rhythm, 3, 862–864. 10.1016/j.hrthm.2005.12.022 16818223

[phy270845-bib-0126] Pepin, M. E. , Konrad, P. J. M. , Nazir, S. , Bazgir, F. , Maack, C. , & Nickel, A. (2025). Mitochondrial NNT promotes diastolic dysfunction in Cardiometabolic HFpEF. Circulation Research, 136, 1564–1578. 10.1161/CIRCRESAHA.125.326154 40340422

[phy270845-bib-0127] Perry, C. J. , & Lawrence, A. J. (2017). Hurdles in basic science translation. Frontiers in Pharmacology, 8, 478. 10.3389/fphar.2017.00478 28769807 PMC5513913

[phy270845-bib-0128] Pfeilschifter, B. , Martinez‐Vilchez, A. , Iqbal, Z. , Potue, P. , Fiegle, D. J. , & Morhenn, K. (2025). Cold storage of mouse hearts prior to cardiomyocyte isolation preserves electromechanical function, microstructure, and gene expression for 24 h. Basic Research in Cardiology, 120, 1055–1074. 10.1007/s00395-025-01131-y 40728729 PMC12518438

[phy270845-bib-0129] Pick, F. , & Krug, N. (2024). Was Deutschland von anderen Ländern lernen kann. Spiegel. https://www.spiegel.de/gesundheit/organspende‐register‐was‐deutschland‐von‐anderen‐laendern‐lernen‐kann‐a‐d1eecb0f‐27d0‐4bc8‐9d58‐2c22bacf469a

[phy270845-bib-0130] Pitoulis, F. G. , Watson, S. A. , Perbellini, F. , & Terracciano, C. M. (2020). Myocardial slices come to age: An intermediate complexity in vitro cardiac model for translational research. Cardiovascular Research, 116, 1275–1287. 10.1093/cvr/cvz341 31868875 PMC7243278

[phy270845-bib-0131] Pogwizd, S. M. , & Bers, D. M. (2008). Rabbit models of heart disease. Drug Discovery Today: Disease Models, 5, 185–193. 10.1016/j.ddmod.2009.02.001 32288771 PMC7105925

[phy270845-bib-0132] Prather, R. S. , Lorson, M. , Ross, J. W. , Whyte, J. J. , & Walters, E. (2013). Genetically engineered pig models for human diseases. Annual Review of Animal Biosciences, 1, 203–219. 10.1146/annurev-animal-031412-103715 25387017 PMC4460601

[phy270845-bib-0133] Prondzynski, M. , Lemoine, M. D. , Zech, A. T. , Horváth, A. , Di Mauro, V. , & Koivumäki, J. T. (2019). Disease modeling of a mutation in α‐actinin 2 guides clinical therapy in hypertrophic cardiomyopathy. EMBO Molecular Medicine, 11, e11115. doi:10.15252/emmm.201911115 31680489 PMC6895603

[phy270845-bib-0134] Protze, S. I. , Liu, J. , Nussinovitch, U. , Ohana, L. , Backx, P. H. , & Gepstein, L. (2017). Sinoatrial node cardiomyocytes derived from human pluripotent cells function as a biological pacemaker. Nature Biotechnology, 35, 56–68. 10.1038/nbt.3745 27941801

[phy270845-bib-0135] Quinn, T. A. , Granite, S. , Allessie, M. A. , Antzelevitch, C. , Bollensdorff, C. , & Bub, G. (2011). Minimum information about a cardiac electrophysiology experiment (MICEE): Standardised reporting for model reproducibility, interoperability, and data sharing. Progress in Biophysics and Molecular Biology, 107, 4–10. 10.1016/j.pbiomolbio.2011.07.001 21745496 PMC3190048

[phy270845-bib-0136] Quinn, T. A. , & Kohl, P. (2013). Combining wet and dry research: Experience with model development for cardiac mechano‐electric structure‐function studies. Cardiovascular Research, 97, 601–611. 10.1093/cvr/cvt003 23334215 PMC3583260

[phy270845-bib-0137] Rao, K. S. , Kameswaran, V. , & Bruneau, B. G. (2022). Modeling congenital heart disease: Lessons from mice, hPSC‐based models, and organoids. Genes & Development, 36, 652–663. 10.1101/gad.349678 35835508 PMC9296004

[phy270845-bib-0138] Rawat, H. , Kornherr, J. , Zawada, D. , Bakhshiyeva, S. , Kupatt, C. , & Laugwitz, K. L. (2023). Recapitulating porcine cardiac development in vitro: From expanded potential stem cell to embryo culture models. Frontiers in Cell and Development Biology, 11, 1111684. 10.3389/fcell.2023.1111684 PMC10227949 37261075

[phy270845-bib-0139] Rebs, S. , Eberl, H. , Wagensohner, N. , Dybkova, N. , Unsöld, J. K. , & Dudek, J. (2025). Enhanced iPSC Cardiomyocyte maturation via combined 3D‐culture and metabolic cues. BioRxiv, 2025.10. 10.1101/2025.10.29.684362

[phy270845-bib-0140] Rechtsinformationssystem des Bundes . (2026). Organtransplantationsgesetz. https://www.ris.bka.gv.at/GeltendeFassung.wxe?Abfrage=Bundesnormen&Gesetzesnummer=20008119

[phy270845-bib-0141] Rees, J. , Winkler, A. , Huettemeister, J. , Stengel, L. , Spangler, P. , & Ramesh, G. (2026). Rostafuroxin ameliorates cardiac glycoside‐induced cardiomyocyte electrolyte imbalances and arrhythmia in ovo. American Journal of Physiology. Heart and Circulatory Physiology, 330(3), H838–H853. 10.1152/ajpheart.00652.2025 41643643

[phy270845-bib-0142] Rehsmann, J. (2023). A revealing scandal: The German transplant scandal between structural failures, moralizing rules, and ambivalent manipulations. Journal of Liver Transplantation, 11, 100168. 10.1016/j.liver.2023.100168

[phy270845-bib-0143] Retsinformation.dk . (2026). Vejledning om samtykke til transplantation fra afdøde personer og til transplantationsrelateret forskning [Guidance on consent for transplantation from deceased persons and for transplantation‐related research], VEJ nr 10009. https://www.retsinformation.dk/eli/retsinfo/2019/10099

[phy270845-bib-0144] Ridder, B. J. , Leishman, D. J. , Bridgland‐Taylor, M. , Samieegohar, M. , Han, X. , & Wu, W. W. (2020). A systematic strategy for estimating hERG block potency and its implications in a new cardiac safety paradigm. Toxicology and Applied Pharmacology, 394, 114961. 10.1016/j.taap.2020.114961 32209365 PMC7166077

[phy270845-bib-0145] Rieblinger, B. , Sid, H. , Duda, D. , Bozoglu, T. , Klinger, R. , & Schlickenrieder, A. (2021). Cas9‐expressing chickens and pigs as resources for genome editing in livestock. Proceedings of the National Academy of Sciences, 118, e2022562118. 10.1073/pnas.2022562118 PMC7958376 33658378

[phy270845-bib-0146] Roney, C. H. , Sim, I. , Yu, J. , Beach, M. , Mehta, A. , & Alonso Solis‐Lemus, J. (2022). Predicting atrial fibrillation recurrence by combining population data and virtual cohorts of patient‐specific left atrial models. Circulation. Arrhythmia and Electrophysiology, 15, e010253. 10.1161/CIRCEP.121.010253 35089057 PMC8845531

[phy270845-bib-0147] Rosshart, S. P. , Herz, J. , Vassallo, B. G. , Hunter, A. , Wall, M. K. , & Badger, J. H. (1979). Laboratory mice born to wild mice have natural microbiota and model human immune responses. Science, 2019, 365. 10.1126/science.aaw4361 PMC7377314 31371577

[phy270845-bib-0148] Sakata, K. , Bradley, R. P. , Prakosa, A. , Yamamoto, C. A. P. , Ali, S. Y. , & Loeffler, S. (2024). Assessing the arrhythmogenic propensity of fibrotic substrate using digital twins to inform a mechanisms‐based atrial fibrillation ablation strategy. Nature Cardiovascular Research, 3, 857–868. 10.1038/s44161-024-00489-x PMC11329066 39157719

[phy270845-bib-0149] Sakata, K. , Yamamoto, C. A. P. , Ali, S. Y. , Loeffler, S. , Prakosa, A. , & Tice, B. M. (2026). Assessment of persistent atrial fibrillation arrhythmogenesis in the right atrium using digital twins. Heart Rhythm, 23, e123–e132. 10.1016/j.hrthm.2025.10.052 41177319

[phy270845-bib-0150] Saleem, U. , van Meer, B. J. , Katili, P. A. , Mohd Yusof, N. A. N. , Mannhardt, I. , & Garcia, A. K. (2020). Blinded, Multicenter evaluation of drug‐induced changes in contractility using human‐induced pluripotent stem cell‐derived Cardiomyocytes. Toxicological Sciences, 176, 103–123. 10.1093/toxsci/kfaa058 32421822 PMC7357169

[phy270845-bib-0151] Salerno, N. , Scalise, M. , Marino, F. , Filardo, A. , Chiefalo, A. , & Panuccio, G. (2023). A mouse model of dilated cardiomyopathy produced by isoproterenol acute exposure followed by 5‐fluorouracil administration. Journal of Cardiovascular Development and Disease, 10, 225. 10.3390/jcdd10060225 37367390 PMC10299031

[phy270845-bib-0152] Schiattarella, G. G. , Altamirano, F. , Tong, D. , French, K. M. , Villalobos, E. , & Kim, S. Y. (2019). Nitrosative stress drives heart failure with preserved ejection fraction. Nature, 568, 351–356. 10.1038/s41586-019-1100-z 30971818 PMC6635957

[phy270845-bib-0153] Schneider, L. V. , Guobin, B. , Methi, A. , Jensen, O. , Schmoll, K. A. , & Setya, M. G. (2023). Bioengineering of a human innervated cardiac muscle model. BioRxiv, 2023.08.18.552653. 10.1101/2023.08.18.552653

[phy270845-bib-0154] Scientific Research . (2026). Ministry of Health welfare and sport donor register. https://english.donorregister.nl/recording‐your‐choise/what‐choices‐do‐you‐have/scientific‐research

[phy270845-bib-0155] Seidel, T. , Fiegle, D. J. , Baur, T. J. , Ritzer, A. , Nay, S. , & Heim, C. (2019). Glucocorticoids preserve the t‐tubular system in ventricular cardiomyocytes by upregulation of autophagic flux. Basic Research in Cardiology, 114, 47. 10.1007/s00395-019-0758-6 31673803 PMC9380897

[phy270845-bib-0156] Selfa Aspiroz, L. , Mennecozzi, M. , Batlle, L. , Corneo, B. , Healy, L. , & Kotter, M. (2025). Promoting the adoption of best practices and standards to enhance quality and reproducibility of stem cell research. Stem Cell Reports, 20, 102531. 10.1016/j.stemcr.2025.102531 40513566 PMC12277813

[phy270845-bib-0157] Shade, J. K. , Prakosa, A. , Popescu, D. M. , Yu, R. , Okada, D. R. , & Chrispin, J. (2021). Predicting risk of sudden cardiac death in patients with cardiac sarcoidosis using multimodality imaging and personalized heart modeling in a multivariable classifier. Science Advances, 7, eabi8020. 10.1126/sciadv.abi8020 34321202 PMC8318376

[phy270845-bib-0158] Shen, C. , Lin, M. , Yaradanakul, A. , Lariccia, V. , Hill, J. A. , & Hilgemann, D. W. (2007). Dual control of cardiac Na+–Ca2+ exchange by PIP2: Analysis of the surface membrane fraction by extracellular cysteine PEGylation. The Journal of Physiology, 582, 1011–1026. 10.1113/jphysiol.2007.132720 17540704 PMC2075243

[phy270845-bib-0159] Shi, R. , Reichardt, M. , Fiegle, D. J. , Küpfer, L. K. , Czajka, T. , & Sun, Z. (2023). Contractility measurements for cardiotoxicity screening with ventricular myocardial slices of pigs. Cardiovascular Research, 119, 2469–2481. 10.1093/cvr/cvad141 37934066 PMC10651213

[phy270845-bib-0160] Sridharan, D. , Pracha, N. , Rana, S. J. , Ahmed, S. , Dewani, A. J. , & Alvi, S. B. (2023). Preclinical large animal porcine models for cardiac regeneration and its clinical translation: Role of hiPSC‐derived Cardiomyocytes. Cells, 12, 1090. 10.3390/cells12071090 37048163 PMC10093073

[phy270845-bib-0161] Statista . (2024). Number of postmortem organ donors in Germany from 1998 to 2023 2024. https://www.statista.com/statistics/1384771/postmortem‐organ‐donors‐germany/

[phy270845-bib-0162] Stauffer, B. L. (2005). Soy diet worsens heart disease in mice. Journal of Clinical Investigation, 116, 209–216. 10.1172/JCI24676 PMC1323247 16395406

[phy270845-bib-0163] Streckfuss‐Bömeke, K. , Tiburcy, M. , Fomin, A. , Luo, X. , Li, W. , & Fischer, C. (2017). Severe DCM phenotype of patient harboring RBM20 mutation S635A can be modeled by patient‐specific induced pluripotent stem cell‐derived cardiomyocytes. Journal of Molecular and Cellular Cardiology, 113, 9–21. 10.1016/j.yjmcc.2017.09.008 28941705

[phy270845-bib-0164] Stüdemann, T. , Rössinger, J. , Manthey, C. , Geertz, B. , Srikantharajah, R. , & von Bibra, C. (2022). Contractile force of transplanted Cardiomyocytes actively supports heart function after injury. Circulation, 146, 1159–1169. 10.1161/CIRCULATIONAHA.122.060124 36073365 PMC9555755

[phy270845-bib-0165] Sun, D. , Gao, W. , Hu, H. , & Zhou, S. (2022). Why 90% of clinical drug development fails and how to improve it? Acta Pharmaceutica Sinica B, 12, 3049–3062. 10.1016/j.apsb.2022.02.002 35865092 PMC9293739

[phy270845-bib-0166] Tan, S. H. , & Ye, L. (2018). Maturation of pluripotent stem cell‐derived Cardiomyocytes: A critical step for drug development and cell therapy. Journal of Cardiovascular Translational Research, 11, 375–392. 10.1007/s12265-018-9801-5 29557052

[phy270845-bib-0167] Tannenbaum, J. , & Bennett, B. T. (2015). Russell and Burch's 3Rs then and now: The need for clarity in definition and purpose. Journal of the American Association for Laboratory Animal Science, 54, 120–132. 25836957 PMC4382615

[phy270845-bib-0168] Thomas, A. , Desai, P. , & Takahashi, N. (2022). Translational research: A patient‐centered approach to bridge the valley of death. Cancer Cell, 40, 565–568. 10.1016/j.ccell.2022.04.014 35561672

[phy270845-bib-0169] Thomas, D. , Cunningham, N. J. , Shenoy, S. , & Wu, J. C. (2022). Human‐induced pluripotent stem cells in cardiovascular research: Current approaches in cardiac differentiation, maturation strategies, and scalable production. Cardiovascular Research, 118, 20–36. 10.1093/cvr/cvab115 33757124 PMC8932155

[phy270845-bib-0170] Tiburcy, M. , Hudson, J. E. , Balfanz, P. , Schlick, S. , Meyer, T. , & Liao, M. L. C. (2017). Defined engineered human myocardium with advanced maturation for applications in heart failure modeling and repair. Circulation, 135, 1832–1847. 10.1161/CIRCULATIONAHA.116.024145 28167635 PMC5501412

[phy270845-bib-0171] Trayanova, N. A. , Lyon, A. , Shade, J. , & Heijman, J. (2024). Computational modeling of cardiac electrophysiology and arrhythmogenesis: Toward clinical translation. Physiological Reviews, 104, 1265–1333. 10.1152/physrev.00017.2023 38153307 PMC11381036

[phy270845-bib-0172] Tsang, H. G. , Rashdan, N. A. , Whitelaw, C. B. A. , Corcoran, B. M. , Summers, K. M. , & MacRae, V. E. (2016). Large animal models of cardiovascular disease. Cell Biochemistry and Function, 34, 113–132. 10.1002/cbf.3173 26914991 PMC4834612

[phy270845-bib-0173] U.S. Department of Health and Human Services . (2018). Assessing the Credibility of Computational Modeling Through Verification and Validation: Application to Medical Devices. American Society of Mechanical Engineers.

[phy270845-bib-0174] Ustaw, D. (2026). Ustawa z dnia 1 lipca 2005 r. o pobieraniu, przechowywaniu i przeszczepianiu komórek, tkanek i narządów [Act of 1 July 2005 on the Collection, Storage, and Transplantation of Cells, Tissues, and Organs.] Dz. U. 2005 No. 169 item 141. https://isap.sejm.gov.pl/isap.nsf/DocDetails.xsp?id=WDU20051691411

[phy270845-bib-0175] van der Geest, J. S. A. , de Boer, T. P. , Terracciano, C. M. , Thum, T. , Dendorfer, A. , & Doevendans, P. A. (2025). Living myocardial slices: Walking the path towards standardization. Cardiovascular Research, 121, 1011–1023. 10.1093/cvr/cvaf079 40354127 PMC12236068

[phy270845-bib-0176] van der Velden, J. , Asselbergs, F. W. , Bakkers, J. , Batkai, S. , Bertrand, L. , & Bezzina, C. R. (2022). Animal models and animal‐free innovations for cardiovascular research: Current status and routes to be explored. Consensus document of the ESC working group on myocardial function and the ESC working group on cellular biology of the heart. Cardiovascular Research, 118, 3016–3051. 10.1093/cvr/cvab370 34999816 PMC9732557

[phy270845-bib-0177] Viceconti, M. , & Emili, L. (2024). Toward Good Simulation Practice. Springer Nature Switzerland. 10.1007/978-3-031-48284-7

[phy270845-bib-0178] Viceconti, M. , Emili, L. , Afshari, P. , Courcelles, E. , Curreli, C. , & Famaey, N. (2021). Possible contexts of use for in Silico trials methodologies: A consensus‐based review. IEEE Journal of Biomedical and Health Informatics, 25, 3977–3982. 10.1109/JBHI.2021.3090469 34161248

[phy270845-bib-0179] Vicente, J. , Zusterzeel, R. , Johannesen, L. , Mason, J. , Sager, P. , & Patel, V. (2018). Mechanistic model‐informed Proarrhythmic risk assessment of drugs: Review of the “CiPA” initiative and Design of a Prospective Clinical Validation Study. Clinical Pharmacology and Therapeutics, 103, 54–66. 10.1002/cpt.896 28986934 PMC5765372

[phy270845-bib-0180] Vornanen, M. , & Hassinen, M. (2016). Zebrafish heart as a model for human cardiac electrophysiology. Channels, 10, 101–110. 10.1080/19336950.2015.1121335 26671745 PMC4960994

[phy270845-bib-0181] Waight, M. C. , Prakosa, A. , Li, A. C. , Truong, A. , Bunce, N. , & Marciniak, A. (2025). Heart digital twins predict features of invasive Reentrant circuits and ablation lesions in scar‐dependent ventricular tachycardia. Circulation. Arrhythmia and Electrophysiology, 18, e013660. 10.1161/CIRCEP.124.013660 40718936 PMC12313252

[phy270845-bib-0182] Wang, G. , McCain, M. L. , Yang, L. , He, A. , Pasqualini, F. S. , & Agarwal, A. (2014). Modeling the mitochondrial cardiomyopathy of Barth syndrome with induced pluripotent stem cell and heart‐on‐chip technologies. Nature Medicine, 20, 616–623. 10.1038/nm.3545 PMC4172922 24813252

[phy270845-bib-0183] Wang, K. , Terrar, D. , Gavaghan, D. J. , Mu‐u‐min, R. , Kohl, P. , & Bollensdorff, C. (2014). Living cardiac tissue slices: An organotypic pseudo two‐dimensional model for cardiac biophysics research. Progress in Biophysics and Molecular Biology, 115, 314–327. 10.1016/j.pbiomolbio.2014.08.006 25124067

[phy270845-bib-0184] Wang, Z. , Tong, C. , Xu, M. , Feng, S. , Dong, M. , & Rao, R. (2026). Optimized methods for the functional cryopreservation of adult human primary cardiomyocytes. Cryobiology, 122, 105583. 10.1016/j.cryobiol.2026.105583 41558336

[phy270845-bib-0185] Watson, S. A. , Duff, J. , Bardi, I. , Zabielska, M. , Atanur, S. S. , & Jabbour, R. J. (2019). Biomimetic electromechanical stimulation to maintain adult myocardial slices in vitro. Nature Communications, 10, 2168. 10.1038/s41467-019-10175-3 PMC6520377 31092830

[phy270845-bib-0186] Welle, D. (2020). German lawmakers reject “opt‐out” organ donor bill. https://www.dw.com/en/german‐parliament‐explicit‐consent‐still‐necessary‐from‐organ‐donors/a‐52022245

[phy270845-bib-0187] Wickramasinghe, N. M. , Sachs, D. , Shewale, B. , Gonzalez, D. M. , Dhanan‐Krishnan, P. , & Torre, D. (2022). PPARdelta activation induces metabolic and contractile maturation of human pluripotent stem cell‐derived cardiomyocytes. Cell Stem Cell, 29, 559–576. 10.1016/j.stem.2022.02.011 35325615 PMC11072853

[phy270845-bib-0188] Wittig, J. G. , & Münsterberg, A. (2020). The chicken as a model organism to study heart development. Cold Spring Harbor Perspectives in Biology, 12, 1–17. 10.1101/cshperspect.a037218 PMC7397825 31767650

[phy270845-bib-0189] Wu, X. , Swanson, K. , Yildirim, Z. , Liu, W. , Liao, R. , & Wu, J. C. (2024). Clinical trials in‐a‐dish for cardiovascular medicine. European Heart Journal, 45, 4275–4290. 10.1093/eurheartj/ehae519 39270727 PMC11491156

[phy270845-bib-0190] Yamamoto, C. , Sakata, K. , Ali, S. Y. , Loeffler, S. , Prakosa, A. , & Tice, B. (2026). Arrhythmogenic substrates in atrial fibrillation and the role of ablation lesions: A longitudinal biatrial digital twin study. Cardiovascular Research, 122, 480–491. 10.1093/cvr/cvag016 41554106 PMC13020427

[phy270845-bib-0191] Yang, D. , Jian, Z. , Tang, C. , Chen, Z. , Zhou, Z. , & Zheng, L. (2024). Zebrafish congenital heart disease models: Opportunities and challenges. International Journal of Molecular Sciences, 25, 5943. 10.3390/ijms25115943 38892128 PMC11172925

[phy270845-bib-0192] Yang, D. , Xu, J. , & Chen, Y. E. (2019). Generation of rabbit models by gene editing nucleases. Methods in Molecular Biology, 1874, 327–345. 10.1007/978-1-4939-8831-0_19 30353523

[phy270845-bib-0193] Yang, H. , Yang, Y. , Kiskin, F. N. , Shen, M. , & Zhang, J. Z. (2023). Recent advances in regulating the proliferation or maturation of human‐induced pluripotent stem cell‐derived cardiomyocytes. Stem Cell Res Ther, 14, 228. 10.1186/s13287-023-03470-w 37649113 PMC10469435

[phy270845-bib-0194] Zaragoza, C. , Gomez‐Guerrero, C. , Martin‐Ventura, J. L. , Blanco‐Colio, L. , Lavin, B. , & Mallavia, B. (2011). Animal models of cardiovascular diseases. Journal of Biomedicine & Biotechnology, 2011, 497841. 10.1155/2011/497841 21403831 PMC3042667

[phy270845-bib-0195] Zhang, K. , Magtibay, K. , Trayanova, N. , & Vigmond, E. (2026). A model of β‐adrenergic stimulation in human ventricular cells for tissue‐scale simulations of sympathetically modulated tachycardias. The Journal of Physiology, 604, 1897–1914. 10.1113/JP289340 41618877 PMC12953024

[phy270845-bib-0196] Zhou, B. , Shi, X. , Tang, X. , Zhao, Q. , Wang, L. , & Yao, F. (2022). Functional isolation, culture and cryopreservation of adult human primary cardiomyocytes. Signal Transduction and Targeted Therapy, 7, 254. 10.1038/s41392-022-01044-5 35882831 PMC9325714

